# Genome and Transcriptome Adaptation Accompanying Emergence of the Definitive Type 2 Host-Restricted *Salmonella enterica* Serovar Typhimurium Pathovar

**DOI:** 10.1128/mBio.00565-13

**Published:** 2013-08-27

**Authors:** Robert A. Kingsley, Sally Kay, Thomas Connor, Lars Barquist, Leanne Sait, Kathryn E. Holt, Karthi Sivaraman, Thomas Wileman, David Goulding, Simon Clare, Christine Hale, Aswin Seshasayee, Simon Harris, Nicholas R. Thomson, Paul Gardner, Wolfgang Rabsch, Paul Wigley, Tom Humphrey, Julian Parkhill, Gordon Dougan

**Affiliations:** The Wellcome Trust Sanger Institute, the Wellcome Trust Genome Campus, Hinxton, Cambridge, United Kingdom^a^; School of Veterinary Sciences, Langford, Bristol, United Kingdom^b^; European Molecular Biology Laboratory Outstation-Hinxton, European Bioinformatics Institute, the Wellcome Trust Genome Campus, Hinxton, Cambridge, United Kingdom^c^; Biomolecular Interaction Centre & School of Biological Sciences, University of Canterbury, Christchurch, New Zealand^d^; National Reference Center for Salmonellae and other Enteric Pathogens, Robert Koch Institut, Wernigerode, Germany^e^; Department of Infection Biology, University of Liverpool, Leahurst Campus, Liverpool, United Kingdom^f^

## Abstract

*Salmonella enterica* serovar Typhimurium definitive type 2 (DT2) is host restricted to *Columba livia* (rock or feral pigeon) but is also closely related to *S*. Typhimurium isolates that circulate in livestock and cause a zoonosis characterized by gastroenteritis in humans. DT2 isolates formed a distinct phylogenetic cluster within *S*. Typhimurium based on whole-genome-sequence polymorphisms. Comparative genome analysis of DT2 94-213 and *S*. Typhimurium SL1344, DT104, and D23580 identified few differences in gene content with the exception of variations within prophages. However, DT2 94-213 harbored 22 pseudogenes that were intact in other closely related *S*. Typhimurium strains. We report a novel *in silico* approach to identify single amino acid substitutions in proteins that have a high probability of a functional impact. One polymorphism identified using this method, a single-residue deletion in the Tar protein, abrogated chemotaxis to aspartate *in vitro*. DT2 94-213 also exhibited an altered transcriptional profile in response to culture at 42°C compared to that of SL1344. Such differentially regulated genes included a number involved in flagellum biosynthesis and motility.

## IMPORTANCE

Whereas *Salmonella enterica* serovar Typhimurium can infect a wide range of animal species, some variants within this serovar exhibit a more limited host range and altered disease potential. Phylogenetic analysis based on whole-genome sequences can identify lineages associated with specific virulence traits, including host adaptation. This study represents one of the first to link pathogen-specific genetic signatures, including coding capacity, genome degradation, and transcriptional responses to host adaptation within a *Salmonella* serovar. We performed comparative genome analysis of reference and pigeon-adapted definitive type 2 (DT2) *S*. Typhimurium isolates alongside phenotypic and transcriptome analyses, to identify genetic signatures linked to host adaptation within the DT2 lineage.

## Introduction

*Salmonella enterica* serovar Typhimurium definitive type 2 (DT2) is highly host restricted to the feral pigeon (*Columba livia*), where it is associated with severe typhoid-like systemic disease (1). The genomic events that accompany the evolution of bacterial pathogens as they adapt to new host species are poorly understood. However, the emergence of host-restricted pathogens from broad-host-range ancestors is a recurring theme in nature and includes many of the major infectious diseases in humans, such as whooping cough (*Bordetella pertussis*) (2), bubonic plague (*Yersinia pestis*) (3, 4), and typhoid (*Salmonella enterica* serotype Typhi) (5, 6). These pathogens emerged relatively recently within broader founder species by restricting host range and pathogenicity. The emergence of bacterial pathogens is an ongoing process as new host niches arise as a result of natural processes or human intervention. The early events of this process remain obscure. *Salmonella enterica* is a paradigm for the study of host adaptation because of the diverse host range and pathogenic potential of the approximately 2,500 serotypes comprising the genus (7). Clinical syndromes caused by serovars of *S. enterica* range from self-limiting diarrhea to invasive diseases, including bacteremia (invasive nontyphoidal *Salmonella* [NTS] disease) and enteric fever (typhoid fever). The serovars of *S. enterica* lie on a spectrum from broad-host-range (promiscuous) serotypes, such as *Salmonella enterica* serovar Typhimurium (mammals and avian), through host-restricted serotypes, such as *S. enterica* serovar Choleraesuis (swine adapted) and *S. enterica* serovar Dublin (bovine adapted), to highly host-restricted serovars, such as *S. enterica* serovar Typhi (human adapted) and *S. enterica* serovar Gallinarum (poultry adapted) (7). Typically, broad-host-range serovars colonize the gut lumen and invade enterocytes of the intestinal mucosa, causing gastroenteritis, but fail to disseminate beyond the lymph nodes, unless the host has an underlying immune defect. Host-restricted serotypes generally exhibit systemic pathogenesis, including dissemination beyond the lymph nodes in immune replete hosts, and exhibit decreased intestinal involvement to an extent that gastroenteritis is no longer a common feature of the disease.

Most *S. enterica* serovars have a relatively broad host range, so the most parsimonious explanation of host-restricted serotype evolution is that they emerged from an ancestor with a broad host range (7). A common approach to study host adaptation is by genome comparison of a broad-host-range (ancestor-like) *Salmonella* serotype with a second serotype that is host restricted: for example, comparison of the prototypical broad-host-range serovar *S.* Typhimurium with the human-restricted serovar *S.* Typhi (5, 6, 8) or the host-restricted serotype *S*. Choleraesuis (9), or comparison of *S. enterica* serovar Enteritidis with the closely related and highly host-restricted pathogen S. Gallinarum (10). Common themes that accompany host adaptation have emerged from these studies, including genome degradation and the formation of pseudogenes. For example, there are ~200 pseudogenes in *S*. Typhi (6) and ~300 pseudogenes in the S. Gallinarum genome (10), with fewer in broad-host-range *S*. Typhimurium isolates, such as SL1344 and DT104 (5). The mechanism by which genome degradation contributes to host adaptation is not known but is likely to include genes required for colonization of alternative host species. Genome comparison of *S*. Typhimurium and *S.* Typhi is complex due to the relatively large degree of genetic divergence. The genomes of typical *Salmonella* serovars differ by over 50,000 single-nucleotide polymorphisms (SNPs), and about 10% of the genes lack an orthologue in pairwise comparison (8).

The epidemiology of *S*. Typhimurium phage types suggested that pathovariants within this serotype ranged from broad host range, such as the multidrug-resistant DT104 pandemic phage types, to the highly host restricted, such as DT2, DT99, and DT56 (1, 11). In order to understand the very early genomic events in the emergence of a pathogen, it is necessary to compare closely related pathogens that nonetheless have distinct host ranges and pathogenicities. Here, we test the hypothesis that genome signatures of host adaptation are associated with changes in pathogenicity consistent with the emergence of the *S*. Typhimurium DT2 group. We describe the high-quality reference genome of *S*. Typhimurium DT2 strain 94-213 and comparative genome analyses of 16 isolates with the reference isolates, including SL1344, for which the whole-genome sequence is available.

## RESULTS

### *S*. Typhimurium DT2 isolates form a distinct phylogenetic clade.

**FIG 1:**
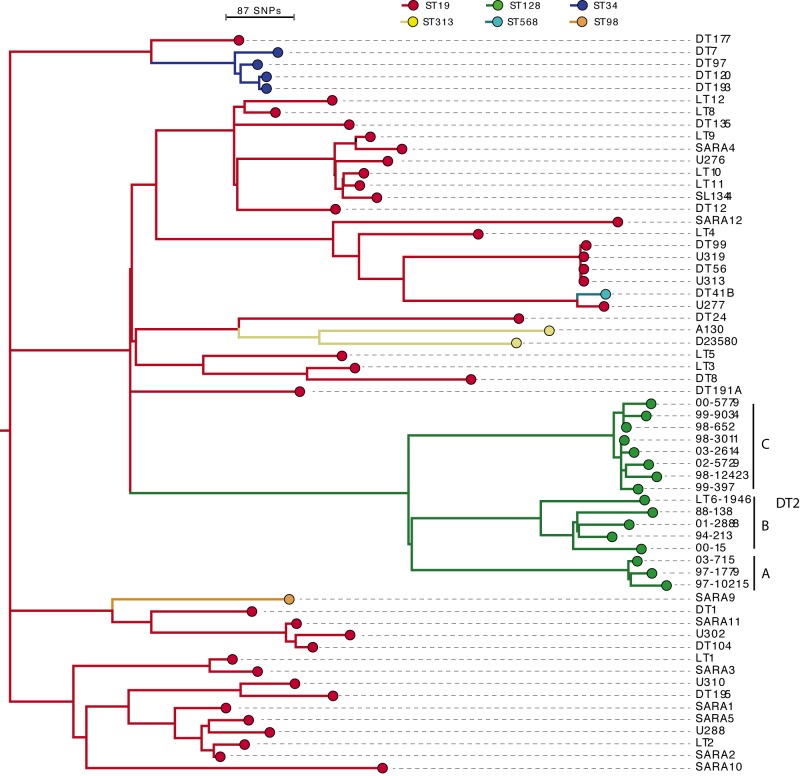
Phylogram of *S*. Typhimurium isolates based on SNPs determined from the whole-genome sequence. A maximum-likelihood tree showing the phylogenetic relationship of *S*. Typhimurium DT2 isolates and a collection of diverse strains from various sources. The length of the scale bar is the estimated number of SNPs determined from the rate of substitution per variable site. MLST groups are color-coded as indicated above the tree. All nodes have >80% bootstrap support unless stated. The sequence type (ST) is indicated: ST19 (red), ST128 (green), ST34 (blue), ST313 (yellow), ST568 (cyan), and ST98 (orange).

*S*. Typhimurium DT2 isolates are highly host restricted to the feral pigeon. Phage type, although informative for epidemiological studies, including outbreak analysis, is not useful in determining the deeper phylogenetic relationship of isolates. Multilocus sequence typing (MLST) indicated that the majority of *S*. Typhimurium isolates are sequence type 19 (ST19) and that this forms the ancestral genotype. At least 19 single- or double-locus MLST variants radiate from the ST19 central hub (12, 13). All *S*. Typhimurium DT2 isolates from pigeon are of ST128, a single-locus variant of the common ST19 (http://mlst.ucc.ie/). In order to determine the high-resolution phylogenetic relationship of DT2 isolates in the context of well-characterized *S*. Typhimurium, the whole-genome sequences of 17 DT2 isolates were determined by Illumina sequencing. These were then placed in the context of a diverse collection of 46 *S*. Typhimurium isolates for which draft genome or reference genome sequences are available, by determining high-confidence single-nucleotide polymorphisms (SNPs) and using these to reconstruct a maximum likelihood phylogenetic tree (Fig. 1). SNPs in repetitive sequences, prophage elements, and other mobile genetic elements were excluded from these analyses. All DT2 isolates were more closely related to one another than they were to any other *S*. Typhimurium isolate in this study, and they formed a distinct clade. In contrast, at least 800 and up to 1,300 SNPs distinguished DT2 isolates from those outside this clade. DT2 isolates cluster into three distinct lineages within the clade, which we designated ST128A, ST128B, and ST128C. These clusters differ by about 500 SNPs, while isolates within each cluster differ by between 10 and 100 SNPs.

### *S*. Typhimurium DT2 exhibits distinct pathogenesis.

We first determined if a representative *S*. Typhimurium DT2 isolate (94-213) had altered pathogenicity in a day-of-hatch chick model. Strain 94-213 was isolated from Neumünster, Germany, in 1994 and has previously been characterized in infection models and comparative genomic hybridization (14). To determine if *S*. Typhimurium strain 94-213 has altered pathogenicity in an avian species, we used 1-week-old chicks as a surrogate infection model (see Fig. S1 in the supplemental material). Two diverse *S*. Typhimurium ST19 isolates, ST4/74 and DT104, colonized the cecum, liver, and spleen of 1-week-old specific-pathogen-free (SPF) chicks by day 7 postinoculation (see Fig. S1). Strain ST4/74 caused mild hepatosplenomegaly and mild “white spot” lesions on the spleen. Most birds infected with DT104 had mild to moderate hepatosplenomegaly and moderate white spot lesions, although one heavily colonized bird had additional blood traces in the cecum. *S*. Typhimurium DT2 94-213 was recovered from the cecum in significantly lower numbers than ST4/74 and DT104. Furthermore, despite colonizing the spleen to a level similar to that of ST4/74 and DT104, 94-213 caused more severe gross pathology typified by moderate hepatosplenomegaly but substantial white spot lesions (data not shown). Strain 94-213 therefore exhibited a phenotype in chicks consistent with host adaptation, reduced colonization of the intestine, and increased severity/pathology at systemic sites.

**FIG 2:**
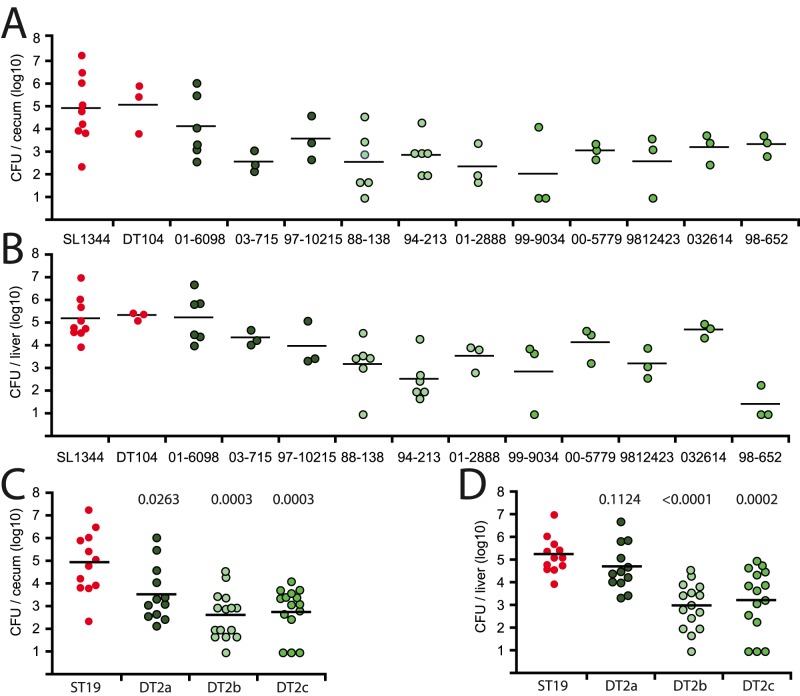
Colonization of the cecum and liver of C57BL/6 mice by *S*. Typhimurium SL1344 and DT104 and representatives of the DT2 lineages. Mice were inoculated with 5 × 10^7^ CFU by the intragastric route with *S*. Typhimurium representing each of the three sublineages of DT2a (dark green), DT2b (light green), and DT2c (medium-green circles) or ST19 isolates, strains SL1344 and DT104 (red circles). Four days postinoculation, the CFU in organ homogenates was determined for the cecum (A) and liver (B). The numbers of CFU per organ in 3 to 9 mice are plotted (circles), and geometric means are indicated (horizontal lines). In order to determine lineage-specific colonization phenotypes, the colonization of the cecum (C) and the liver (D) for isolates from each lineage were plotted together (circles), and the geometric means are indicated. The probability that the colonization of isolates from each DT2 sublineage is significantly different from that of SL1344 and DT104 was calculated using an unpaired Mann-Whitney test. The *P* value is indicated above the plots for the DT2a, DT2b, and DT2c sublineages.

A common feature of host adaptation by bacterial pathogens is the loss of virulence for secondary hosts. We therefore determined the virulence in mice of two *S*. Typhimurium isolates from ST19 (SL1344 and DT104) and eleven DT2 isolates representing the diversity of this clade (three from DT2A, three from DT2B, and five from DT2C) (Fig. 2). Groups of mice were inoculated orally with 5 × 10^7^ CFU of each isolate (Fig. 2A and B), and to determine lineage-specific phenotypes, data from mice inoculated with isolates from each DT2 sublineage were combined for analysis (Fig. 2C and D). Colonization of the cecum (Fig. 2C) and liver (Fig. 2D) was greater for both ST19 isolates tested than for the DT2 ones from clusters DT2B and DT2C. ST19 isolates also colonized the cecum to a greater extent than isolates of DT2A, but those from cluster DT2A colonized the liver to a level similar to those of the two ST19 isolates (Fig. 2). These data indicated that evolution of the DT2 lineage involved genetic changes that resulted in attenuation in colonization of the murine cecum and, in the case of lineages DT2B and DT2C, also the liver in the murine host.

### Comparative genomics of DT2 94-213 and SL1344.

To study relationships between the genomes of DT2 and a representative ST19 *S*. Typhimurium isolate in detail, the sequence of *S*. Typhimurium DT2 94-213 was determined to the reference genome standard (15). The DT2 94-213 genome consisted of a single circular chromosome of 4,814,400 nucleotides and a plasmid of 93,844 nucleotides that is highly related to the pSLT plasmid of LT2 (5). Comparison of the chromosome of 94-213 to those of *S*. Typhimurium LT2, DT104, SL1344, and D23580 did not reveal any evidence of DNA inversions, translocations, or duplications. However, a total of 692 *S*. Typhimurium DT2 lineage-specific SNPs were identified. This represents the greatest level of lineage-specific divergence within the *S*. Typhimurium clade described to date. D23580, a representative of ST313 that is associated mainly with invasive NTS disease in sub-Saharan Africa, harbors 553 specific SNPs (13). SL1344, LT2, and DT104, representatives of ST19 that are generally associated with sporadic and outbreak gastroenteritis worldwide, have 409, 236, and 497 isolate-specific SNPs, respectively (5).

A total of 95 genes in SL1344 did not have an orthologue in DT2 94-213. All of these, except five genes (SL1485 to SL1489), were carried on prophage elements; DT2 94-213 lacks the Fels-2 prophage and contains a region of variation in a Gifsy-1-like prophage (see Fig. S2 in the supplemental material). Three genes carried by DT2 94-213 did not have orthologues in SL1344 and were also associated with a region of variation within Gifsy-1-like prophage. Perhaps surprisingly, no DT2 94-213-specific genes or prophage elements were present. That genes SL1485 to SL1489 of SL1344 do not have orthologues in DT2 94-213 was previously reported from microarray experiments (14). Orthologues of these genes were present in all other DT2 strains sequenced in this study, consistent with previous findings that this deletion is specific to strain 94-213 (14).

### *S*. Typhimurium DT2 94-213 exhibits an altered coding capacity in the chromosome and plasmid pSLT.

94-213 harbors a total of 84 pseudogenes, 21 of which were intact in *S*. Typhimurium LT2, SL1344, DT104, and D23580 (Table 1) (5, 13). However, just two of these DT2-associated pseudogenes, *pcgL* and *fepE*, have previously been implicated in host-pathogen interactions (16, 17). PcgL is a periplasmic d-alanyl-d-alanine dipeptidase and is involved in peptidoglycan metabolism. This gene confers the ability to use d-alanyl-d-alanine as a sole source of carbon, and inactivation results in hypervirulence in the murine model of salmonellosis, due to an as-yet-undefined impact of d-alanyl-d-alanine on the host innate immune response (17). FepE is a regulator of the O-antigen chain length of lipopolysaccharide (LPS) and has been implicated in *Salmonella* survival in the inflamed intestine when bile levels are elevated (18). Of the other pseudogenes, the *cigR* gene is present on *Salmonella* pathogenicity island 3 (SPI-3) and encodes a putative inner membrane protein that has not previously been implicated in host-pathogen interaction (19, 20). Four genes that are pseudogenes in SL1344 were potentially functional in 94-213, as they are present as extended open reading frames due to small indels. The *nfsA* gene (STM_DT2_08501), an oxygen-insensitive NADPH nitroreductase, is a pseudogene in SL1344 (orthologue SL0850). Mutation of *nfsA* results in resistance to nitrofuran antibiotics (21), which is widespread in clinical and veterinary *Salmonella* isolates. That this is a functional gene in DT2 is consistent with the restriction of this isolate to wild-bird populations that are likely to not have been exposed to selection by nitrofuran antibiotics. The other three genes, STM_DT2_36591, STM_DT2_28151, and STM_DT2_34091, are of unknown function.

**TABLE 1:** Pseudogenes in *S*. Typhimurium 94-213 that are functional in strains SL1344, DT104, and D23580

Systematic ID	Common name	SL1344 orthologue	Pseudogene in^*a*^:			Annotation
			97-10215	03-715	98-652	
DT2_03371		SL0336	Y	Y	N	Putative inner membrane protein, regulator
DT2_04541	*cof*	SL0451	Y	Y	Y	Putative hydrolase
DT2_05801	*fepE*	SL0577	N	N	N	Ferric enterobactin/enterochelin transporter
DT2_06161	*dpiB*	SL0613	Y	Y	Y	Two-component regulatory system
DT2_08721	*ltaA*	SL0873	N	N	N	l-Allo-threonine aldolase
DT2_09191	*ycaI*	SL0920	N	N	N	Putative recombination protein
DT2_10901	*yceA*	SL1093	N	N	N	Hypothetical protein
DT2_14831		SL1490	Y	Y	Y	Putative 1,4-alpha-glucan-branching enzyme (glgB)
DT2_15221	*pcgL*	SL1530	Y	Y	Y	d-Alanyl-d-alanine dipeptidase for peptidoglycan
DT2_17401	*slp*	SL1748	Y	Y	Y	Putative outer membrane, pseudogene in ST313
DT2_21071		SL2110	Y	Y	Y	Putative inner membrane protein
DT2_24911		SL2492	N	N	N	Putative anaerobic dimethyl sulfoxide reductase
DT2_26381		SL2663	Y	Y	Y	Putative ABC transporter transmembrane region
DT2_30271		SL3107	Y	Y	Y	Putative amidohydrolase
DT2_31181	*ygjU*	SL3198	N	N	N	Putative dicarboxylate permease
DT2_32241	*yhcG*	SL3304	Y	Y	Y	Putative cytoplasmic protein
DT2_33671	*yhfL*	SL3447	Y	N	Y	Putative outer membrane lipoprotein
DT2_35271	*Tag*	SL3608	N	N	N	3-Methyl-adenine DNA glycosylase I
DT2_36451	*cigR*	SL3727	N	N	N	Putative inner membrane protein
DT2_40991	*aphA*	SL4185	Y	Y	Y	Nonspecific acid phosphatase/phosphotransferase
DT2_43031	*mgtA*	SL4387	Y	Y	Y	Mg^2^-ATPase transporter

^a^ Y, yes; N, no.

*S*. Typhimurium 94-213 plasmid pSLT_DT2 is 95 bp shorter than pSLT_LT2 due to 5 deletions ranging from 1 to 81 bp. There are also 24 SNP differences, of which eight are nonsynonymous (see Table S2). Of note is the D325N substitution in SpvB, an ADP-ribosyl transferase involved in intracellular survival in macrophages (22, 23). There is also a 1-bp deletion in the upstream region of *spvR*, the regulator of the *spv* operon, that may impact on the Shine-Dalgarno sequence. Another feature of note is the presence of multiple polymorphisms in the *traD* gene. This gene contains three nonsynonymous SNPs and an in-frame deletion of 81 bp. These polymorphisms may affect the conjugal transfer of pSLT_DT2.

### DT2 lineage-specific pseudogenes do not individually result in attenuation in the murine typhoid model.

**FIG 3:**
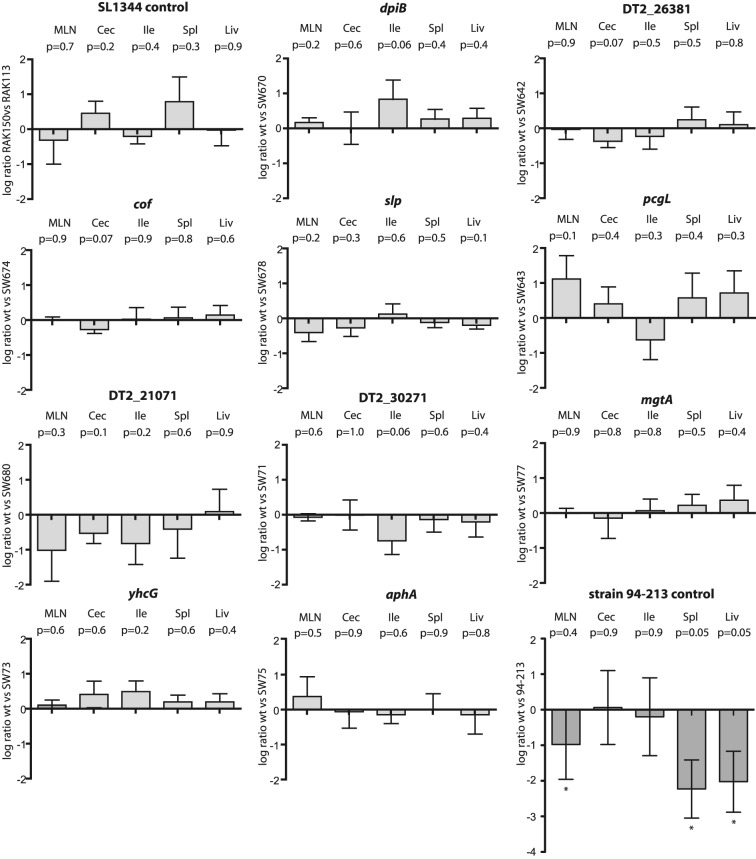
Mixed-inoculum competitive infection of C57BL/6 mice to compare virulence of *S*. Typhimurium RAK113 and SL1344 containing deletion of pseudogenes in 94-213. An equal mixture (log_10_ ratio = 0) containing approximately 1 × 10^7^ CFU of RAK113 and test strain was inoculated orally. Filled bars indicate the log_10_ ratio ± standard errors (SE) of CFU (RAK113/test strain) on day five postinoculation in organ homogenates: mesenteric lymph nodes (MLN), cecum (Cec), ileum (Ile), spleen (Spl), and liver (Liv). *, Student’s *t* test was used to determine if the output log_10_ ratio was significantly different from the input one.

In order to determine the phenotypic effect of pseudogenes that were introduced into the ancestral DT2 lineage and are therefore shared by all extant DT2 isolates (Table 1), we replaced the *S*. Typhimurium SL1344 orthologous genes of DT2_03371, DT2_04541 (*cof*), DT2_06161 (*dpiB*), DT2_14831, DT2_15221 (*pdgL*), DT2_17401 (*slp*), DT2_21071, DT2_26381, DT2_30271, DT2_32241 (*yhcG*), DT2_40991 (*aph*), and DT2_43031 (*mgtA*) with an *aph* (aminoglycoside transferase, kanamycin resistance) gene by allelic exchange. The ability of these derivatives to compete with a wild-type SL1344 for colonization of the mouse following intragastric inoculation was determined in mixed-inoculum experiments. None of the mutant derivatives exhibited a decreased ability to compete with the wild type for colonization of the mouse intestine or deeper organs (Fig. 3).

### Evidence for functional genome degradation due to nonsynonymous SNPs in 94-213.

**FIG 4:**
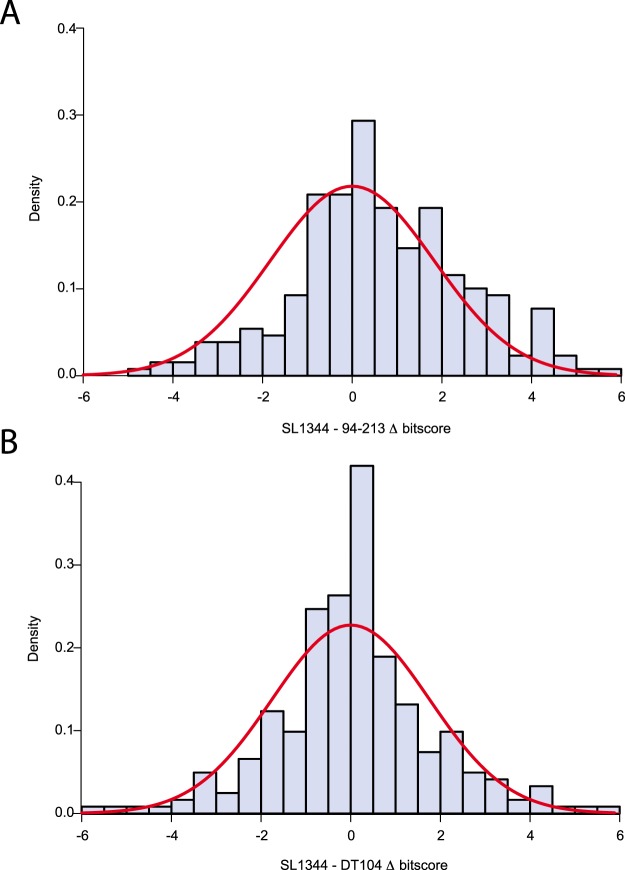
Frequency distribution of Δbit scores calculated from a Pfam scan of 94-213 and DT104 peptides compared with SL1344 orthologues that contain amino acid substitutions. The bit score of HMM domains determined using a Pfam scan were measured, and the differences between SL1344/94-213 and SL1344/DT104 were calculated. The frequency distributions for SL1344/94-213 (A) and SL1344/DT104 (B) are plotted as bars. The normal distribution, centered around a Δbit score of 0, was calculated using the variation observed in the calculated data.

A total of 484 nonsynonymous SNPs were present in 408 genes in 94-213 relative to SL1344. In order to estimate amino acid substitutions that have an impact on protein function, we developed a novel approach comparing the peptide sequence in orthologue pairs from DT2 94-213 and SL1344 that contained one or more substitutions with protein families in the Pfam database. This generated a score (bit score) for each hidden Markov model (HMM) match that reflects how well the query sequence matches the model for the HMM family. The difference in bit score for 94-213 and SL1344 orthologue HMM hits (Δbit score) was calculated, and the frequency distribution was plotted (Fig. 4A). We considered that the greater the Δbit score, the greater the likelihood is of a functional impact due to the polymorphism. A total of 614 Pfam domains defined by HMMs were identified in the 408 polymorphic proteins, since some proteins contained more than one domain (see Table S2). The mean of the Δbit score distribution for the 94-213 and SL1344 comparison was shifted from 0 with an overrepresentation of positive Δbit score (a lower Δbit score in 94-213 than in SL1344; one-sample *t* test, *P* = 1.7 × 10^−5^). This is consistent with a larger-than-expected proportion of amino acid substitutions in 94-213 resulting in a peptide sequence with a lower match to the Pfam HMM than would be expected by chance. We performed a similar analysis of nonsynonymous SNPs in *S*. Typhimurium DT104 and SL1344, which exhibit a similar level of divergence but are both host generalists. A total of 386 genes contained nonsynonymous SNPs in DT104 relative to SL1344, and 514 HMM domains were identified in these proteins. The mean of the Δbit score distribution of DT104 and SL1344, unlike the 94-213 and SL1344 comparison, was not significantly different from 0 (Fig. 4B). A number of proteins involved in host-pathogen interactions exhibited large differences in bit score in 94-213 and SL1344 (see Table S2). These included the chaperone component of two usher chaperone family fimbriae, *stdC* and *bcfG*, and the *sipD* protein (V152A substitution). The *tar* gene that encodes a methyl-accepting chemotaxis protein that is required for chemotaxis response to aspartic acid contained polymorphisms in two Pfam HMM domains, a codon deletion resulting in deletion of A98 and an SNP resulting in the V400A substitution.

**FIG 5:**
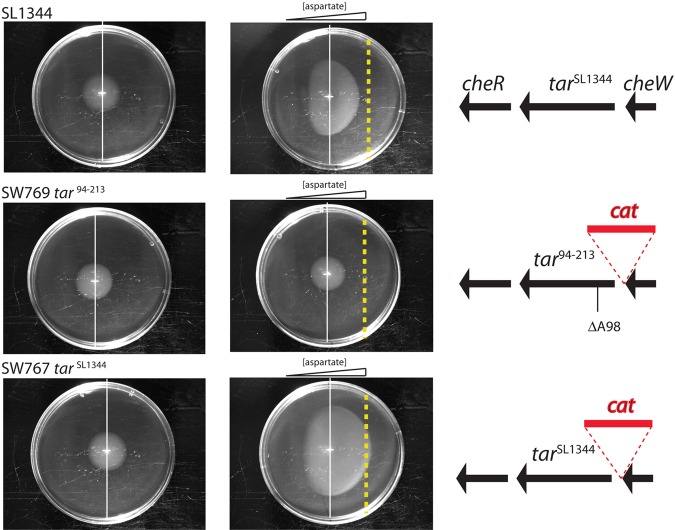
Polymorphisms in the Tar protein of *S*. Typhimurium DT2 strain 94-213 results in loss of function of the methyl-accepting chemotaxis protein. The *tar* gene of strain 94-213 was transferred to strain SL1344 using bacteriophage P22-mediated transduction by selecting for the cotransfer of a *cat* gene inserted in the 5′ region of the *tar* promoter. The SW769 transductant, that cotransduced the ΔA98 and V400A polymorphisms, no longer responded by chemotaxis toward aspartate in the agar (dashed yellow line), while the SW767 transductant, in which the *cat* gene but not the tar polymorphisms were transferred, responded to aspartate in a similar fashion to SL1344.

Together, these observations raised the possibility that the accumulation of some nonsynonymous SNPs in 94-213 might result in impaired function of the encoded peptides. To test this hypothesis directly, we determined the impact on function of the polymorphisms in *tar*, one of the genes in which the encoded protein exhibited the greatest Δbit score. We transferred the *tar* gene of strain 94-213 (*tar*^94-213^) into SL1344 by cotransduction with a *cat* gene inserted in the 5′ region of the *tar* promoter. The recombinant SL1344 derivative that harbored the *tar*^94-213^ gene, unlike the SL1344 parent strain, did not migrate toward a source of aspartic acid in 0.3% agar (Fig. 5). This was not due to insertion of the *cat* gene in the 5′ region of the *tar* promoter, since a recombinant derivative that contained the *cat* gene but had the *tar*^SL1344^ genotype exhibited chemotaxis toward aspartic acid. These data provided direct evidence that a single amino acid deletion contributed to functional degradation of the coding capacity and phenotypic change during the evolution of the DT2 pathogen.

### *S*. Typhimurium DT2 isolate 94-213 remodels its transcriptome in response to elevated temperature.

The relatively small number of genomic features that distinguished DT2 and SL1344 was striking, considering the distinct pathogenicities and epidemiologies of these pathogens. We therefore addressed the hypothesis that adaptation of the transcriptome of 94-213 has characteristics that may contribute to a lifestyle within the avian host. One key distinguishing feature of the mammalian host and the avian host is body temperature, 37°C and 42°C, respectively. We therefore determined whether temperature is a signal used by the pathogen to alter gene expression patterns in such a manner that may favor a particular disease outcome. We compared the expression profiles of *S*. Typhimurium 94-213 and SL1344 at 37°C and 42°C in mid-log-phase planktonic culture in order to determine genes that are differentially expressed specifically in response to elevated temperature.

**FIG 6:**
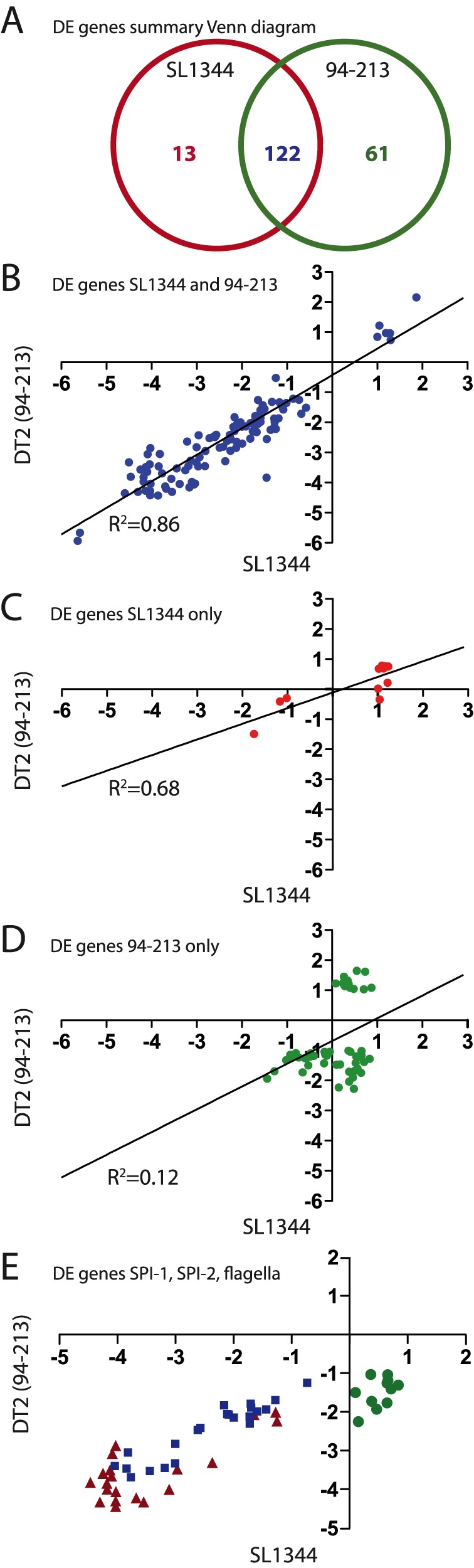
Comparison of genes differentially expressed at 42°C and 37°C in *S*. Typhimurium DT2 strain 94-213 and SL1344. (A) Venn diagram summarizing the numbers of genes differentially expressed in 94-213 and SL1344, SL1344 only, and 94-213 only. The log_2_-fold changes in expression at 42°C compared to that at 37°C of genes that were significantly differentially expressed are plotted for strain 94-213 (*y* axis) and SL1344 (*x* axis). (A) Venn diagram summarizing the number of genes that were differentially expressed (DE): differentially expressed in 94-213 and SL1344 (B), SL1344 only (C), and 94-213 only (D). (E) Differentially expressed type associated with the SPI-1 (red triangles), SPI-2 (blue squares), or flagellum biosynthesis, motility, and chemotaxis (green circles).

**FIG 7:**
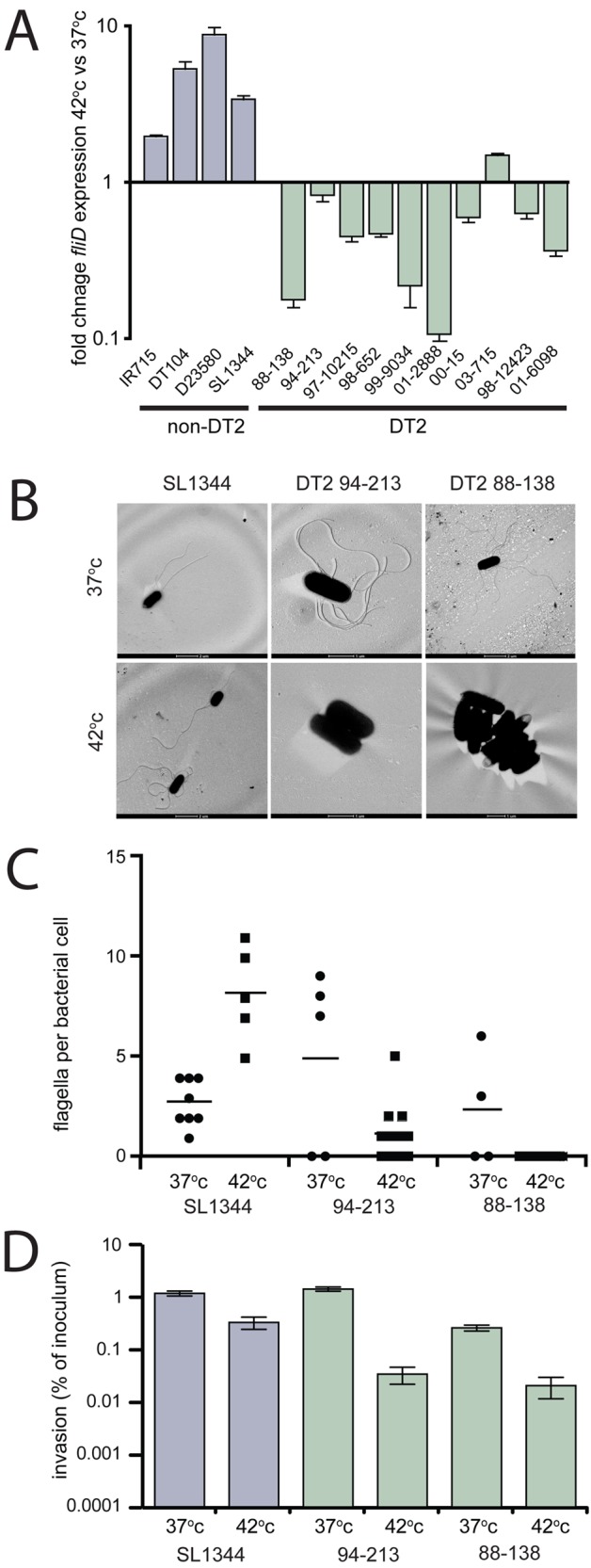
Flagellum gene expression, elaboration of flagella, and invasion of enterocytes. (A) Relative expression of the *fliD* gene determined by quantitative RT-PCR of four strains from outside the DT2 clade and 10 DT2 isolates during culture at 42°C and 37°C. (B) Transmission electron micrograph of negatively stained *S*. Typhimurium cultured at 42°C or 37°C showing expression of flagella. (C) Enumeration of flagella associated with negatively stained *S*. Typhimurium in random TEM fields. (D) Invasion of cultured T84 enterocyte-like cells by *S*. Typhimurium strains following culture of the inoculum at 42°C or 37°C determined using a gentamicin protection assay. The number of CFU recovered 2 h postinoculation is expressed as the percentage of the initial inoculum. The mean percentages and standard errors from five biological replicates are indicated. Data from a representative of duplicate experiments are shown.

**FIG 8:**
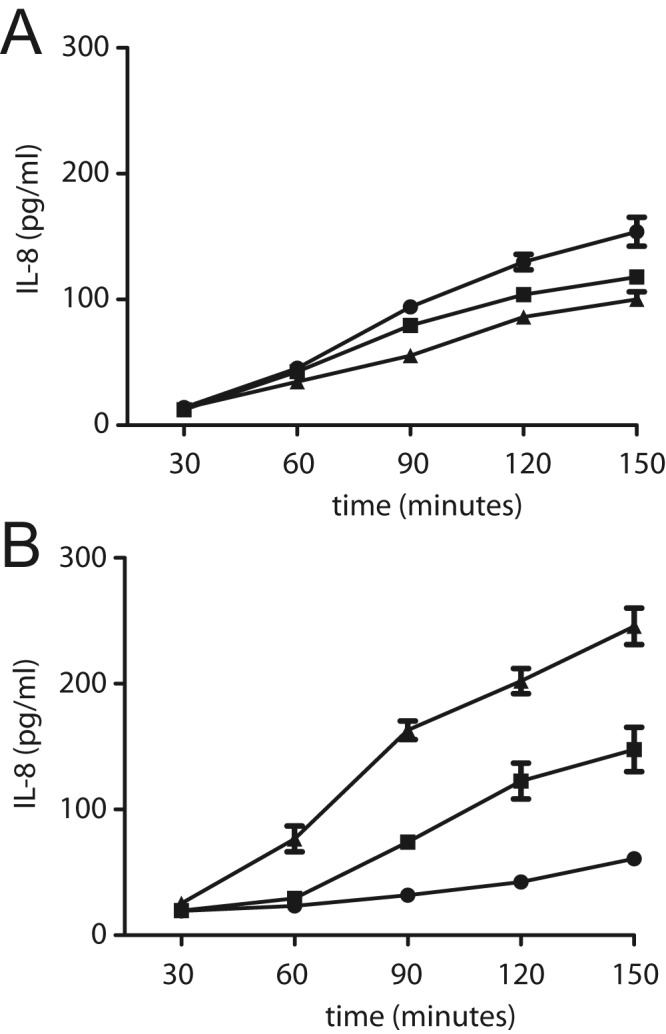
Impact of temperature of *S*. Typhimurium culture on interaction with host enterocytes in culture at 37°C and 42°C. Presence of the proinflammatory cytokine IL-8 in the supernatant of cultured T84 enterocyte-like cells after exposure to *S*. Typhimurium strains following culture of the inoculum at 37°C (A) or 42°C (B). The mean concentration of IL-8 (pg/ml) in the tissue culture medium supernatant ± standard deviation from three replicate wells infected with SL1344 (triangle), 94-213 (square), and 88-138 (circle).

A total of 196 genes were differentially regulated at 42°C relative to 37°C in SL1344 or 94-213 or both during planktonic culture in a rich medium (Fig. 6; see also Table S3 in the supplemental material). A total of 122 were differentially regulated in both SL1344 and 94-213, of which most were downregulated at 42°C (99 genes), and the change in expression showed a high degree of correlation in SL1344 and 94-213 (*R*^2^ = 0.86). These genes are likely to represent the ancestral response to elevated temperature and included 41 encoded on SPI-1 or SPI-2 or ones associated with the type III secretion systems (TTSSs) encoded on these islands. The few genes with increased expression at the elevated temperature included those encoding the well-characterized heat shock and thermotolerance-associated response proteins, GroES and GroEL. Only 13 genes were significantly differentially regulated in SL1344 alone, but nonetheless the relative expression in the two strains showed considerable correlation (*R*^2^ = 0.68). In contrast, 61 genes were differentially regulated at the elevated temperature in DT2 94-213, suggesting that this isolate has rewired regulation of a portion of its genome in order to respond to elevated temperature to influence interactions with the avian host. Strikingly, 11 of 56 genes expressed at a lower level at the elevated temperature in DT2 94-213 are involved in motility, chemotaxis, or flagella biosynthesis (Fig. 6E). Decreased expression of *fliD* following culture at 42°C was confirmed in a number of DT2 isolates by using quantitative reverse transcription-PCR (qRT-PCR), but no such decrease in expression of this gene was observed for a number of *S*. Typhimurium isolates from outside the DT2 clade (SL1344, DT104, D23580, and IR715) (Fig. 7A). Flagella are of critical importance in host-pathogen interactions, for motility and chemotaxis (24, 25), and because the flagellin monomer is a key pathogen-associated molecular pattern (PAMP) that engages the host innate immune system through Toll-like receptor 5 (TLR5) and Ipaf (26–29). Determination of the presence of flagella by transmission electron microscopy (TEM) with negative staining indicated that SL1344 elaborated similar numbers of flagella during culture at 42°C and at 37°C. However, consistent with the transcription data, 94-213 had significantly fewer flagella per cell following culture at 42°C than at 37°C (Fig. 7B and C). The decreased expression of flagella was even more pronounced in 88-138; indeed, none were observed on the surface at the elevated temperature. The abilities of SL1344 and DT2 94-213 and 88-138 to invade cultured epithelium-like T84 cells following culture at either 37°C or 42°C differed significantly. In each case, 0.3 to 1% of the initial inoculum was intracellular 2 h after infection when cultured at 37°C (Fig. 7D). However, culture of SL1344 at 42°C resulted in a modest decrease in invasion of ~3-fold, while 94-213 and 88-138 exhibited a considerably greater decrease (10- to 30-fold). These data are consistent with a general decrease in invasiveness of all isolates at elevated culture temperature, which is likely to be associated with decreased expression of SPI-1 genes at 42°C compared with 37°C (Fig. 7E). The reason for the dramatic decrease in invasion of T-84 cells observed specifically in DT2 isolates is not likely to be solely due to decreased flagella biosynthesis, motility, and chemotaxis genes, since bacteria were centrifuged onto the T84 cells. Culture of *S*. Typhimurium at the elevated temperature does impact on the subsequent outcome of interaction with epithelial-like cells in culture. Production of the proinflammatory cytokine interleukin 8 (IL-8) by T84 cells was similar in response to strain SL1344 and to the two DT2 strains 94-213 and 88-138 when the inoculum was cultured at 37°C (Fig. 8). In contrast, following culture of the *S*. Typhimurium inoculum at 42°C, the IL-8 response was considerably lower in the two DT2 strains than in SL1344. This was consistent with specific adaptation of the DT2 strains to evasion of detection by the host innate immune system.

## DISCUSSION

*S*. Typhimurium DT2 isolates are remarkable in their host adaptation to pigeon, where they cause a severe paratyphoid-like disease in young birds, but are rarely isolated from cases of gastroenteritis in humans or other animals. Phylogenetic analysis using high-quality SNPs from the core genome of diverse *S*. Typhimurium isolates and 17 DT2 isolates indicated that DT2 formed a discrete clade within the *S*. Typhimurium phylogenetic tree. Further, this clade harbors three distinct sublineages (DT2A, DT2B, and DT2C) that exhibited lineage-specific virulence traits in the genetically susceptible murine typhoid model. While isolates from all three DT2 lineages colonized the murine cecum significantly less than SL1344, isolates from lineage DT2a colonized the liver to a similar level. This revealed a potentially complex evolutionary history for which the most parsimonious explanation is that genomic changes resulted in modulation of virulence before divergence of the three DT2 sublineages. Molecular changes revealed an attenuation in colonization of the liver that may have occurred independently on the DT2b and DT2c lineages.

A previous microarray analysis between DT2 94-213 and LT2 concluded that they share virtually all of their genes (14). Here, high-quality genome sequence analysis of *S*. Typhimurium DT2 94-213 identified only a few DT2-specific genes. Further, just 692 DT2 lineage-specific SNPs, defined as those not present in the complete genome sequences of the SL1344 (30), D23580 (13), or DT104 genome, were identified. Differences in gene repertoire were almost entirely due to the absence of Fels-2 from the DT2 genome and a region of variation in a prophage related to Gifsy-1 of SL1344. None of these differences in prophage repertoire impacted on known cargo genes involved in host-pathogen interactions (31). The paucity of a DT2-specific sequence was perhaps surprising considering the profound differences in epidemiology, pathogenicity, and host range exhibited by DT2 and contrasts with more divergent pathogens, such as *S.* Typhi, that contain a number of differences with closely related pathogens, including large deletions and insertions, such as SPI-7 (6). These observations suggest that the early event in the formation of a new host-restricted pathogen does not necessarily involve acquisition of new genetic material.

A significant form of genome degradation observed in 94-213 was the accumulation of pseudogenes, with a total of 22 lineage-specific pseudogenes present in the 94-213 genome. This was considerably more specific pseudogenes than observed in SL1344 (6 pseudogenes) and was a number similar to that in D23580 (23 pseudogenes), an ST313 lineage invasive NTS disease isolate with considerable genome degradation that might be associated with host adaptation (13). A lack of virulence gene degradation associated with host-restricted pathogens was previously noted for other *Salmonella* serotypes, including *S*. Typhi (6) and S. Gallinarum (10). This is perhaps not surprising if we consider that many central virulence determinants, such as the invasion locus carried on SPI-1 and the intracellular survival locus carried by SPI-2, were gained by *S. enterica* before or during the formation of the species and therefore were likely involved in mechanisms of pathogenesis critical to all *Salmonella* serotypes and pathovariants regardless of host range.

We determined the pseudogenes that are common to all DT2 lineages, as these are candidate genes whose functions were lost during the early events in the formation of the DT2 pathogen cluster. Inspection of orthologous genes in 97-10215, a representative of lineage DT2a, and 98-652, a representative of lineage DT2c, which are pseudogenes in 94-213, indicated that 12 pseudogenes were formed in the ancestral DT2 lineage. Individual mutation of orthologous genes in SL1344 did not, on their own, result in decreased colonization of the murine cecum or liver. This is perhaps surprising, since pseudogene formation is commonly thought to contribute to restriction of host range in adapted pathogens. It remains possible that a combination of genome degradation of several of these genes resulted in the decrease in ability of DT2 isolates to colonize the murine intestine or that factors other than coding capacity account for this phenotype.

Loss of coding capacity reported in host-restricted bacterial pathogens has generally been in the form of large deletions or pseudogenes (4, 13, 32). However, more subtle changes may also affect function, but these are difficult to unambiguously determine from sequence alone. We used a novel approach to identify candidate functionally significant substitutions or small deletions in protein sequence, based on sequence alignment with the HMM profiles housed by the Pfam database. Differences in the bit score for each pairwise comparison of orthologous genes highlighted proteins that had diverged from canonical members of an HMM protein domain family. We carried out pairwise comparison of three finished genomes of *S*. Typhimurium that exhibited similar degrees of divergence from one another. We found that in the frequency distribution of Δbit scores of the host-restricted DT2 94-213 in combination with the broad host-range SL1344, there was a shift from a distribution centered on 0 toward positive Δbit scores, consistent with functional genome degradation in DT2 strain 94-213. This shift was not observed in a comparison of Δbit scores of two broad-host-range strains. This approach may be useful as a general tool to identify functionally significant polymorphisms in related genomes. Here, we provide proof of principle by investigating the impact of a polymorphism that resulted in a relatively large reduction in the bit score in the *S*. Typhimurium DT2 protein. Deletion of an alanine residue in the methyl-accepting chemotaxis protein Tar resulted in the loss of the ability of *S*. Typhimurium to sense and move along a gradient of aspartate. This may reflect the relative loss of intestinal disease exhibited by *S*. Typhimurium DT2 since chemotaxis has previously been implicated in enteropathogenesis (25) and the ability to gain access to nutrients and host-derived electron acceptors for respiration (33).

The finding that strain 94-213 has a distinct transcriptional response to culture at 42°C relative to strain SL1344 suggested that this might be an important adaptation to the avian host. A large proportion of the genes that were differentially regulated specifically in the DT2 strain were those associated with flagellum biosynthesis, motility, and chemotaxis. Flagella are critical to the interaction of *Salmonella* with host cells through multiple functions. They are surface-localized appendages that function in motility and chemotaxis, contributing to invasion by increasing the likelihood of contact (34, 35). In addition, flagellin monomer functions as a pathogen-associated molecular pattern (PAMP) through its interaction with TLR5 (26) and interleukin-1β (IL-1β)-converting enzyme-activating factor (IPAF) (27, 28). Furthermore, the importance of flagella in host-adapted disease was recently underlined by the observation that the expression of TLR11, which recognizes flagellin, is a barrier to infectivity of *S*. Typhi in mice. *S*. Typhi causes a severe invasive disease in TLR11 knockout mice and in humans in which TLR11 is not expressed due to a nonsense mutation (36). Decreased expression of flagella at the body temperature of the avian host (>40°C) is consistent with this being an adaptation to evade detection by the host immune system. Acquisition of the *tviA* gene by *S*. Typhi integrated the regulation of flagella into the OmpR and RcsB regulons, such that flagellum expression is decreased following invasion of the intestinal mucosa, thereby avoiding detection by TLR5 present on the basolateral surface of enterocytes and CD11c^+^ dendritic cells in the lamina (37–39). Furthermore, introduction of the S. Typhi *tviA* gene into *S*. Typhimurium resulted in decreased expression of flagella in low-salt medium and also resulted in increased invasiveness in experimental infections of chicken (40). We have found that an *S*. Typhimurium DT2 strain decreases expression of flagella by a mechanism that is independent of *tviA*. Indeed, no candidate regulator gene was present in DT2 strain 94-213 that was not also present in strain SL1344, a strain that did not exhibit downregulation of flagella at the elevated temperature. It is therefore likely that as-yet-unidentified nucleotide polymorphisms account for the divergence in regulation of flagella and other genes between DT2 strain 94-213 and SL1344. The downregulation of flagella expression is potentially highly significant to the pathogenicity of DT2 isolates, as this is a recurring theme in the pathoadaptation of avian-associated serotypes of *S. enterica* (41, 42).

Together, our data represent a first insight into the events that occur during the formation of a new host-restricted pathogen. The genomic events accompanying the epidemiological and pathogenicity changes reported for *S*. Typhimurium DT2 are not due to large-scale acquisition of new virulence genes, since virtually no outside prophage elements were present in a representative genome sequence finished to a high standard. This suggests that at least in the case of pathogens such as *Salmonella* that have a broad array of virulence genes, adaptation to new hosts may occur by subtle adaptations to an existing virulence gene repertoire, in the form of genome degradation of polymorphisms or by rewiring of regulatory networks.

## MATERIALS AND METHODS

### Strains and culture conditions.

Isolates of *S*. Typhimurium DT2 (00-5779, 99-9034, 98-652, 98-3011, 03-2614, 02-5729, 98-12423, 99-397, 88-138, 01-2888, 94-213, 00-15, 03-715, 97-1779, 97-10215) have been described previously (14). Other *S*. Typhimurium field isolates used in determination of phylogeny have been described previously (43). Bacteria were routinely cultured aerobically at 37°C in Luria-Bertani (LB) broth or LB with 1% agar. Where appropriate, antibiotics were supplemented as follows: chloramphenicol, 0.03 mg/ml (LB-Cm); kanamycin, 0.05 mg/ml (LB-Km); or nalidixic acid, 0.05 mg/ml (LB-Nal).

### Construction of *S*. Typhimurium recombinant strains.

Mutants of *S*. Typhimurium SL1344 or DT2 wild isolates were made using the Red recombinase system, based on methods described previously (44). *S*. Typhimurium was transformed with pSIM18 plasmid carrying the Red recombinase genes (45). For allelic exchange with the *cat* or *aph* genes, pKD3 or pKD4 were used for PCR amplification with cycle conditions of 94°C for 30 s, 30 cycles of 94°C for 30 s, 57°C for 30 s, and 72°C for 2 min, and a final elongation at 72°C for 5 min. For deletion of genes by allelic replacement, oligonucleotide primers were designed containing 45 nucleotides of sequence with identity to immediately upstream of the ATG start and downstream of the predicted stop codon. These sequences directed the precise deletion of the target gene. In addition, following the gene-specific sequence, each forward oligonucleotide primer contained the sequence 5′ GTGTAGGCTGGAGCTGCTTCG 3′, and each reverse primer contained the sequence 5′ CATATGAATATCCTCCTTAG 3′, that prime at sites flanking the antibiotic resistance cassettes in pKD3 and pKD4. These primers were used to PCR amplify the *cat* or the *aph* gene from pKD3 or pKD4, respectively. A total of 3 to 5 µg of PCR product was transformed into *S*. Typhimurium strain SL1344/pSIM18 by electroporation, and transformants in which the target gene had been replaced with the resistance gene cassette were selected on LB-Cm or LB-Km agar. The resistance gene marker was transduced into *S*. Typhimurium SL1344. This last step resulted in the transfer of the mutant locus into a fresh genetic background that had undergone minimal manipulation in order to reduce the likelihood of mutations unlinked to the target mutation that may have arisen during the mutagenesis process, complicating the analysis. This methodology gave rise to the SW670 (Δ*dpiB*::*aph*), SW642 (Δ*DT2*_*26381*::*aph*), SW674 (Δ*cof*::*aph*), SW678 (Δ*slp*::*aph*), SW643 (Δ*pcgL*::*aph*), SW680 (Δ*DT2*_*21071*::*aph*), SW71 (Δ*DT2*_*30271*::*aph*), SW643 (Δ*mgtA*::*aph*), SW73 (Δ*yhcG*::*aph*), and SW75 (Δ*aphA*::*aph*) strains. In addition, two control *S*. Typhimurium strains were constructed by this approach in which the *phoN* gene of strain SL1344 was replaced by the *cat* gene, encoding resistance to chloramphenicol (RAK113). A strain in which *phoN* was replaced by the *aph* gene, encoding resistance to kanamycin, was constructed by P22-mediated transduction of Δ*phoN*::*aph* from *S*. Typhimurium strain AJB715 (46) into strain SL1344. The *phoN* gene has previously been shown to have no impact on murine infection (46), and therefore these strains were used as the wild type in mixed-inoculum experiments. For transfer of the *tar*^94-213^ gene into SL1344, a chloramphenicol acetyltransferase gene (*cat*) was introduced into the *S*. Typhimurium DT2 genome *xbp* downstream of the stop codon of *cheW* using the primers 5′ AGGCACTCTCACCGCTGGCGGAAGCATAACGGTGAATATTGCCGGGTGTAGGCTGGAGCTGCTTCG 3′ and 5′ GCATCACACGTCGCGTAATAACGTTGCCGGATGGCGTCGCGCCATCATATGAATATCCTCCTTAG 3′, giving rise to strain SW767. The *cat* gene in this strain was transduced into SL1344 by using bacteriophage P22, giving rise to strain SW769. Cotransduction of polymorphisms in the *tar* locus following amplification by PCR primers flanking the two polymorphic sites was determined by sequence determination by Sanger technology using primers 5′ TATTGCCATTGTCCAGAAACTG 3′, 5′ GTTATTTCTAACGAATTACGTCAG 3′, 5′ ATATCCGTGACGCGCGTAAC 3′, and 5′ GTCGACGGCGTAGTAAACAC 3′.

### Transmission electron microscopy.

Bacteria were cultured in LB broth, and 0.1 ml was harvested by centrifugation, washed in 1 ml distilled water (dH_2_O), and resuspended in 1 ml dH_2_O. A total of 2 to 5 µl of this bacterial suspension was applied to a freshly glow-discharged carbon/Formvar-coated copper grid, allowing time for the bacteria to settle for 1 min before drawing the liquid across the grid surface with a piece of cut filter paper. Dried samples were gently washed with double-distilled water, and the grids were transferred to a BAE 250 coating unit. The bacteria were shadowed at an angle of 20° with platinum to a deposit thickness of 1 to 2 nm and imaged on a 120-kV FEI Spirit BioTwin using an *F*_4.15_ Tietz charge-coupled device (CCD).

### Tissue culture and determination of IL-8 production.

T84 cells (ECACC catalogue number 88021101) were cultured in Ham’s F-12--Dulbecco's modified Eagle’s medium (DMEM) (1:1) supplemented with 2 mM l-glutamine and 10% heat-inactivated fetal bovine serum (FBS). For bacterial invasion assays, the cells were trypsinized and plated into 24-well costar plates at 1 × 10^5^ cells/ml and 0.5 ml/well, 24 h prior to the assay. Following infection at a multiplicity of infection (MOI) of 10, the cells were left at 37°C for 30 min, and the medium was then replaced with that containing 50 µg/ml gentamycin. At the time points indicated in Fig. 8, supernatants were removed, filtered through 20-µm-pore-size filters, and stored at −80°C for cytokine assays. The remaining cells were lysed with 1% Triton in phosphate-buffered saline (PBS) for enumeration of viable bacterial CFU.

The supernatants were investigated for IL-8 content utilizing the Becton Dickinson human IL-8 CBA Flex set and the human soluble protein buffer master kit as per the manufacturer’s instructions. All reactions were analyzed on a Becton Dickinson FACsAria11 using FCAP software to generate IL-8 content in values of picograms per milliliter.

### Animal infections.

All animal procedures were performed in accordance with the United Kingdom Home Office Inspectorate under the Animals (Scientific Procedures) Act 1986. The Wellcome Trust Sanger Institute Ethical Review Committee granted ethical approval for these procedures.

In all mouse experiments, female, 7- to 8-week-old C57BL/6 mice (Charles River) were inoculated by gavage with *S*. Typhimurium suspended in PBS, pH 7.4. For mixed-inoculum experiments, in order to distinguish the wild-type strain from the mutant test strains, a *cat* (chloramphenicol acetyltransferase, chloramphenicol resistance) gene was inserted in the *S*. Typhimurium SL1344 chromosome in a position that has previously been described to have no effect on colonization of the murine host (46, 47) (*phoN* locus, strain RAK113). Five mice were inoculated orally with a 1:1 ratio [log_10_ = 0] of approximately 1 × 10^7^ CFU of strain RAK113 (SL1344 Δ*phoN*::*cat*) and the test strain. When mice were moribund (<80% body weight compared with body weight on the day of inoculation) or on day five postinoculation, mice were culled and the numbers of CFU of each strain in homogenized mesenteric lymph nodes (MLN), cecum, ileum, spleen, and liver was determined by serial dilution in PBS, pH 7.4, by culture on LB agar containing chloramphenicol and LB agar containing kanamycin. For single-inoculum experiments of field isolates, groups of three to nine 6- to 8-week-old female C57BL/6 mice were inoculated with approximately 1 × 10^8^ CFU of each test strain, and 4 days postinoculation, mice were culled and the cecum and liver were recovered and homogenized. Serial 10-fold dilutions were plated on LB-Cm or LB-Km agar, as appropriate, to determine CFU per organ. The ratio of the wild type (strain RAK113) to the test strain was transformed to a log_10_ value, and the two-tailed Student *t* test in the Prism 4 software version 4.0c (Graph Pad) was used to determine if these values were significantly different from the input ratio (*P* values of <0.05 were considered significantly different).

For experimental infections of chicken, SPF Rhode Island Red chicks were obtained from the Pirbright Institute, Compton Laboratory (Newbury, United Kingdom). Chicks were housed in individual infection groups and reared on the floor at a temperature of 30°C with *ad libitum* access to water and a vegetable protein-based diet (SDS, Witham, United Kingdom). At 7 days of age, birds were infected by oral gavage with approximately 2 × 10^8^ CFU of each isolate in a volume of 0.2 ml LB broth grown for 18 h from pure colonies in a shaking incubator at 37°C.

At 3 days postinfection, chicks were killed by neck dislocation, and liver and spleen were removed aseptically to quantify bacterial load. Both ceca were removed, and contents were squeezed into a sterile container. Tissues and cecal content were homogenized in a Seward Biomaster 80 stomacher (Seward, Worthing, United Kingdom), diluted in PBS, pH 7.4, and then plated onto modified brilliant green agar plates (Oxoid, Basingstoke, United Kingdom) as serial 10-fold dilutions to determine CFU of *Salmonella* per gram of tissue or cecal content.

### Sequencing and bioinformatics.

*S*. Typhimurium strain 94-213 was fragmented by sonication, and libraries were generated by using the pUC18 plasmid vector, using size fractions ranging from 1.0 to 2.5 kb. Sequence reads were analyzed, giving a theoretical coverage of 10×. Insert libraries were sequenced using dye terminator chemistry on ABI3700 automated sequencers. The sequence was assembled, finished, and annotated as described previously (48), using Artemis (49) to collate data and facilitate annotation. Read alignment and SNP detection used paired-end Illumina sequence data mapped to the reference genome *S*. Typhimurium strain SL1344 (50) by using SMALT (ftp://ftp.sanger.ac.uk/pub4/resources/software/smalt). SNPs were identified using SAMtools mpileup and filtered with a minimum mapping quality of 30 and quality ratio cutoff of 0.75. SNPs in prophage sequences and repetitive regions of the *S*. Typhimurium reference strain SL1344 (accession no. FQ312003; 1054795 to 1100036, 2039803 to 2079890, 2726717 to 2777229, 2815382 to 2825915, 2855616 to 2888522, 2890073 to 2900377, 3099171 to 3100233, 3116271 to 3117792, 3434695 to 3435556, and 1915772 to 1915863) were excluded from further analysis. A maximum-likelihood phylogenetic tree was constructed from the SNP alignment with RAxML version 7.0.4 (51) using a general time-reversible (GTR) substitution model with gamma correction for among-site rate variation. Support for nodes on the trees was assessed using 100 bootstrap replicates.

The frequency distribution of HMM Δbit scores (SL1344 Δbit score − query strain Δbit score) was determined for peptide sequences from strains SL1344, 94-213, and DT104. The expected normal distribution was calculated using the standard deviation of observed Δbit score values after excluding outliers.

### RNA preparation and transcription analysis.

For microarray and qRT-PCR, *S*. Typhimurium was cultured in LB broth to an optical density at 600 nm (OD_600_) of 0.6, and RNA was immediately stabilized by the addition of 20 ml of RNAlater (Qiagen) to 10 ml of culture. RNA was prepared using the RNeasy minikit (Qiagen), and DNA was removed using RNase-free DNase I (Qiagen). A custom Agilent array was designed (design accession) using standard Agilent protocols. A total of 50 ng of total RNA was amplified and labeled with cyanine 3-CTP by following the manufacturer’s protocol (Agilent low-input Quick Amp WT labeling kit, one color, product no. 5190-2943; Agilent). Labeling efficiency was assessed using the NanoDrop-8000 spectrophotometer (Thermo Scientific). Cy-3-labeled cRNA was hybridized onto Agilent custom *S*. Typhimurium microarrays for 17 h at 65°C. After hybridization, the microarray slides were washed and scanned using the Agilent DNA high-resolution microarray scanner (Agilent; G2505C) by following the manufacturer’s protocol. Data from the Agilent array were analyzed using the Agilent Feature Extraction (AFE) software (version 10.1). Array features were calculated using AFE default settings for the GE2-v5_10_Apr08 protocol. The analysis was performed using scripts written in R (version 2.11.1 [31 May 2010]). Using the application available from the Bioconductor’s LIMMA library, http://www.bioconductor.org/packages/release/bioc/html/limma.html, a linear model fit was applied to the data that was generated. Top differentially expressed genes were tabulated for each contrast using the method of Benjamini and Hochberg to correct the *P* values (52). The lower the adjusted *P* value, the more significant the change in gene expression (the more in accordance the replicate beads on the arrays). Adjusted *P* and log-fold change values are strongly correlated but not absolutely so. The adjusted *P* value of <0.05 was used in the microarrays to indicate significant difference. A positive log_2_-fold change indicated upregulation in the first stated sample.

For qRT-PCR, RNA samples (40 µg) were DNase I (Thermo Scientific) treated in a 100-µl volume and diluted to 100 ng/µl. RNA samples were reverse transcribed and used as the template for real-time PCR with Express one-step SYBR GreenER (Invitrogen) in a 20-µl total reaction volume. Real-time PCR was performed using a StepOnePlus real-time PCR system (Applied Biosystems) with gene-specific oligonucleotides. Data were analyzed by using the comparative cycle threshold (*C*_*T*_) method, where the target gene transcription of each sample was normalized to the *C*_*T*_ of the *waaY* transcript.

### Nucleotide sequence and microarray accession numbers.

The sequence for strain 94-213 can be found at EMBL accession no. HG326213. The sequences of 15 additional *S*. Typhimurium DT2 strains were determined using Genome Analyzer (Illumina) sequencing of multiplexed genomic DNA libraries using standard protocols. The raw sequence data for these strains can be found with the following accession numbers: 97-10215, ERR037576; 98-652, ERR037576; 99-397, ERR037578; 00-5779, ERR037583; 02-5729, ERR037574; 03-2614, ERR037575; 99-9034, ERR028070; 03-715, ERR028071; 01-2888, ERR028072; 00-15, ERR028073; 98-12423, ERR028077; 97-1797, ERR028067; and 98-3011, ERR028069. Microarray data were submitted to the NCBI GEO database under accession no. GSE48061.

## SUPPLEMENTAL MATERIAL

Figure S1 Colonization of 1-week-old chicks by *S*. Typhimurium isolates. Groups of five 1-week-old chicks were inoculated with 10^8^ CFU of *S*. Typhimurium. The geometric means ± standard deviations of CFU recovered from cecum, liver, and spleen organ homogenates are plotted. Download Figure S1, PDF file, 0.3 MB

Figure S2 Whole-genome comparison of *S*. Typhimurium strain SL1344, DT2 strain 94-213, DT104, LT2, and D23580. BLASTN comparison of *S*. Typhimurium strains for which finished reference sequence assembly is available, viewed using the Artemis comparison tool (ACT). Red and blue bands represent the forward and reverse matches, with the intensity of the color indicating the percentage identity of the match. Download Figure S2, PDF file, 0.4 MB

Table S1 Alignments. Table S1, XLSX file, 0.1 MB.

Table S2 Gene annotations. Table S2, DOCX file, 0.2 MB.

Table S3 Gene annotations. Table S3, XLSX file, 0.1 MB.
