# Supplementary figures and images for: Genome and Transcriptome Adaptation Accompanying Emergence of the Definitive Type 2 Host-Restricted Salmonella enterica Serovar Typhimurium Pathovar
Source: mBio. 2013 Aug 27;4(5):e00565-13. doi: 10.1128/mBio.00565-13 (PMC3760250; doi:10.1128/mBio.00565-13)

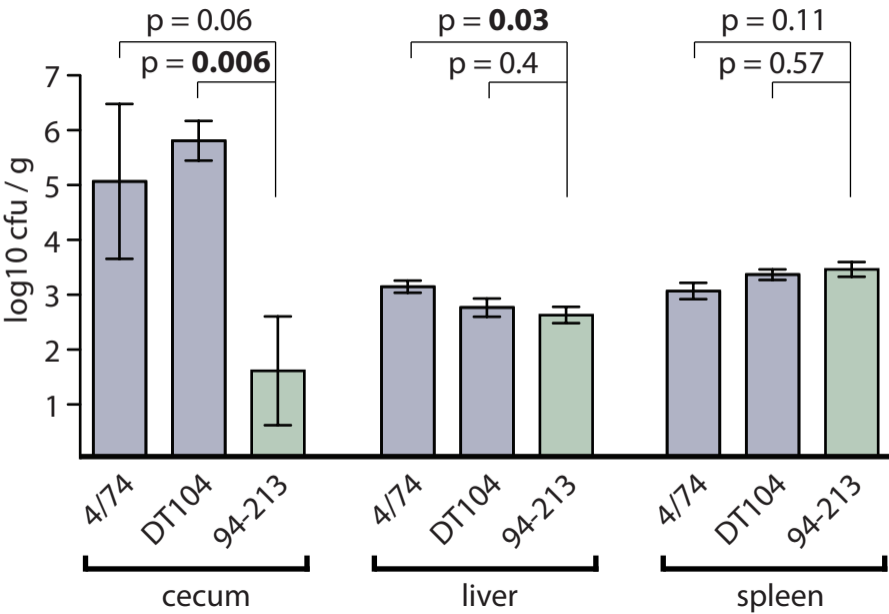

Supplement: Figure S1 — Colonization of 1-week-old chicks by S. Typhimurium isolates. Groups of five 1-week-old chicks were inoculated with 108 CFU of S. Typhimurium. The geometric means ± standard deviations of CFU recovered from cecum, liver, and spleen organ homogenates are plotted. Download [file mbo004131612sf01.pdf]

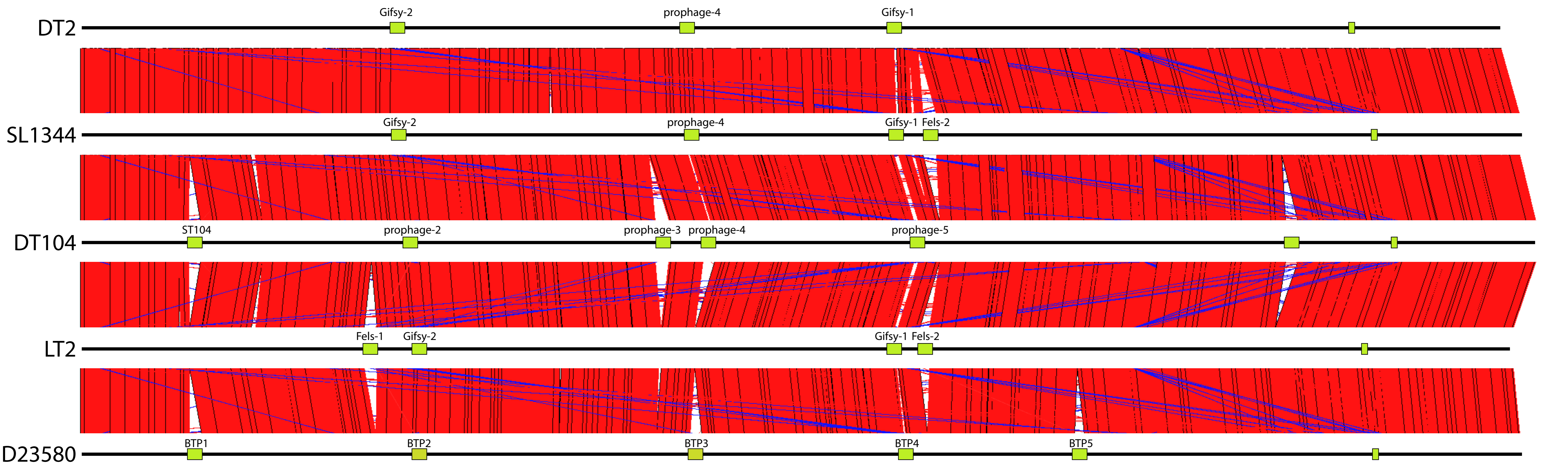

Supplement: Figure S2 — Whole-genome comparison of S. Typhimurium strain SL1344, DT2 strain 94-213, DT104, LT2, and D23580. BLASTN comparison of S. Typhimurium strains for which finished reference sequence assembly is available, viewed using the Artemis comparison tool (ACT). Red and blue bands represent the forward and reverse matches, with the intensity of the color indicating the percentage identity of the match. Download [file mbo004131612sf02.pdf]
