# Supplementary material for: Genome and Transcriptome Adaptation Accompanying Emergence of the Definitive Type 2 Host-Restricted Salmonella enterica Serovar Typhimurium Pathovar
Source: mBio. 2013 Aug 27;4(5):e00565-13. doi: 10.1128/mBio.00565-13 (PMC3760250; doi:10.1128/mBio.00565-13)
Supplement: Table S2 — Gene annotations. [file mbo004131612st2.docx]

| Systematic_ID | Gene | SL1344 orthologue | Annotation | Δ bit-score | HMM accession |
| --- | --- | --- | --- | --- | --- |
| STM_DT2_04921 | - | SL0489 | conserved hypothetical protein | 9.5 | PF07446.4 |
| STM_DT2_18481 | tar | SL1854 | methyl-accepting chemotaxis protein II | 8.3 | PF02203.8 |
| STM_DT2_29261 | stdC | SL3006 | probable fimbrial chaperone protein | 6.8 | PF02753.10 |
| STM_DT2_11401 | - | SL1142 | putative protein kinase C inhibitor | 5.9 | PF01230.16 |
| STM_DT2_25751 | - | SL2576 | Phage lysozyme | 5.3 | PF00959.12 |
| STM_DT2_21621 | - | SL2162 | putative membrane protein | 4.7 | PF10808.1 |
| STM_DT2_15371 | - | SL1544 | putative phosphotransferase enzyme | 4.7 | PF03611.7 |
| STM_DT2_43981 | - | SL4481 | ferric iron reductase protein | 4.6 | PF06276.5 |
| STM_DT2_00271 | bcfG | SL0027 | fimbrial chaperone | 4.5 | PF02753.10 |
| STM_DT2_16641 | - | SL1671 | conserved hypothetical protein | 4.5 | PF04269.5 |
| STM_DT2_32301 | nanT | SL3310 | putative sialic acid transporter | 4.5 | PF07690.9 |
| STM_DT2_04581 | mdlB | SL0455 | putative ABC transporter ATP-binding | 4.4 | PF00664.16 |
| STM_DT2_21841 | setB | SL2184 | sugar efflux transporter | 4.4 | PF07690.9 |
| STM_DT2_18481 | tar | SL1854 | methyl-accepting chemotaxis protein II | 4.2 | PF00015.14 |
| STM_DT2_43891 | mdoB | SL4472 | putative phosphoglycerol transferase | 4.2 | PF00884.16 |
| STM_DT2_25011 | hscB | SL2502 | Co-chaperone protein hscB | 4.1 | PF07743.6 |
| STM_DT2_05631 | - | SL0560 | similar to a putative phospohtase iso | 4.1 | PF01380.15 |
| STM_DT2_12891 | - | SL1290 | Putative acyl-CoA dehydrogenase | 4.1 | PF02771.9 |
| STM_DT2_39921 | trmA | SL4078 | tRNA (uracil-5)-methyltransferase | 4 | PF05958.4 |
| STM_DT2_41551 | dcuS | SL4241 | two-component sensor kinase | 3.9 | PF02518.19 |
| STM_DT2_25511 | - | SL2552 | similar to a DNA recombinase | 3.6 | PF08400.3 |
| STM_DT2_27821 | sipD | SL2862 | pathogenicity island 1 effector protein | 3.5 | PF06511.4 |
| STM_DT2_16451 | - | SL1652 | conserved hypothetical protein | 3.4 | PF01300.11 |
| STM_DT2_43151 | - | SL4399 | ornithine carbamoyltransferase | 3.4 | PF02729.14 |
| STM_DT2_07271 | ybgC | SL0726 | conserved hypothetical protein | 3.4 | PF03061.15 |
| STM_DT2_09821 | - | SL0985 | putative phage minor tail protein | 3.4 | PF00877.12 |
| STM_DT2_30181 | - | SL3098 | LysR-family transcriptional regulator | 3.4 | PF00324.14 |
| STM_DT2_17491 | - | SL1757 | putative membrane protein | 3.3 | PF00571.21 |
| STM_DT2_37011 | torA | SL3788 | trimethylamine-N-oxide reductase precursor | 3.3 | PF00384.15 |
| STM_DT2_09971 | - | SL1000 | conserved hypothetical protein | 3.2 | PF03476.9 |
| STM_DT2_35611 | sgbE | SL3642 | putative sugar isomerase | 3.2 | PF00596.14 |
| STM_DT2_35631 | - | SL3644 | conserved hypothetical protein | 3.2 | PF07944.5 |
| STM_DT2_34331 | - | SL3513 | putative kinase/trancriptional regulatory protein | 3.1 | PF00294.17 |
| STM_DT2_06321 | ybeA | SL0629 | conserved hypothetical protein | 3 | PF02590.10 |
| STM_DT2_07971 | rhlE | SL0796 | putative ATP-dependent RNA helicase rhlE | 3 | PF00271.24 |
| STM_DT2_33121 | rplF | SL3392 | 50S ribosomal subunit protein L6 | 2.9 | PF00347.16 |
| STM_DT2_35521 | - | SL3633 | putative carboxylic acid dehydrogenase | 2.9 | PF02615.7 |
| STM_DT2_02051 | clcA | SL0204 | H | 2.8 | PF00654.13 |
| STM_DT2_24501 | purC | SL2450 | phosphoribosylaminoimidazole-succinocarboxamide synthase | 2.8 | PF01259.11 |
| STM_DT2_13761 | - | SL1378 | conserved hypothetical protein | 2.8 | PF03702.7 |
| STM_DT2_06891 | kdpB | SL0687 | potassium-transporting ATPase B chain | 2.7 | PF00122.13 |
| STM_DT2_42931 | - | SL4377 | conserved hypothetical protein | 2.7 | PF07071.4 |
| STM_DT2_03161 | yafA | SL0314 | conserved hypothetical protein | 2.6 | PF06500.4 |
| STM_DT2_41181 | - | SL4204 | putative xanthine/uracil permeases family protein | 2.6 | PF00860.13 |
| STM_DT2_25521 | - | SL2553 | prophge protein | 2.6 | PF12421.1 |
| STM_DT2_24441 | acrD | SL2444 | putative efflux pump | 2.6 | PF00873.12 |
| STM_DT2_33001 | yhdN | SL3380 | conserved hypothetical protein | 2.5 | PF09350.3 |
| STM_DT2_25521 | - | SL2553 | prophge protein | 2.4 | PF09327.4 |
| STM_DT2_36771 | - | SL3759 | putative carbohydrate kinase | 2.4 | PF00294.17 |
| STM_DT2_32431 | - | SL3323 | oxaloacetate decarboxylase beta chain | 2.4 | PF03977.6 |
| STM_DT2_35851 | gpsA | SL3666 | glycerol-3-phosphate dehydrogenase | 2.4 | PF07479.7 |
| STM_DT2_42761 | - | SL4361 | putative carbohydrate kinase | 2.3 | PF00294.17 |
| STM_DT2_33691 | gph | SL3449 | phosphoglycolate phosphatase | 2.3 | PF00702.19 |
| STM_DT2_26961 | gabD | SL2775 | succinate-semialdehyde dehydrogenase | 2.2 | PF00171.15 |
| STM_DT2_38431 | tatC | SL3929 | sec-independent protein translocase protein | 2.2 | PF00902.11 |
| STM_DT2_11771 | msgA | SL1180 | putative virulence protein (MsgA) | 2.2 | PF06183.6 |
| STM_DT2_39471 | fpr | SL4033 | ferredoxin--NADP reductase | 2.2 | PF00175.14 |
| STM_DT2_19291 | - | SL1932 | putative phage protein | 2.2 | PF04865.7 |
| STM_DT2_37271 | yidY | SL3814 | putative membrane transport protein | 2.2 | PF07690.9 |
| STM_DT2_00561 | - | SL0056 | putative glutaconyl-CoA decarboxylase | 2.1 | PF02436.11 |
| STM_DT2_42151 | rnr | SL4301 | ribonuclease R (RNase R) | 2.1 | PF00773.12 |
| STM_DT2_01641 | ygbK | SL0163 | conserved hypothetical protein | 2 | PF07005.4 |
| STM_DT2_08561 | potI | SL0857 | putrescine transport system permease | 2 | PF00528.15 |
| STM_DT2_28591 | rumA | SL2938 | 23S rRNA | 2 | PF01938.13 |
| STM_DT2_34021 | malT | SL3482 | MalT regulatory protein | 2 | PF00196.12 |
| STM_DT2_04711 | acrB | SL0468 | acriflavin resistance protein B | 1.9 | PF00873.12 |
| STM_DT2_41271 | nrfA | SL4213 | cytochrome c552, subunit of nitrite reductase complex | 1.9 | PF02335.8 |
| STM_DT2_29021 | ptsP | SL2981 | phosphoenolpyruvate-protein phosphotransferase | 1.9 | PF02896.11 |
| STM_DT2_21701 | folE | SL2170 | GTP cyclohydrolase I | 1.9 | PF01227.15 |
| STM_DT2_32961 | sapG | SL3376 | potassium transport protein | 1.9 | PF02254.11 |
| STM_DT2_13991 | rstB | SL1402 | two component sensor kinase | 1.9 | PF02518.19 |
| STM_DT2_26951 | ygaF | SL2774 | putative GAB DTP gene cluster repressor | 1.9 | PF01266.17 |
| STM_DT2_33611 | nirB | SL3441 | nitrite reductase large subunit | 1.8 | PF07992.7 |
| STM_DT2_39631 | metB | SL4049 | cystathionine gamma-synthase | 1.8 | PF01053.13 |
| STM_DT2_15481 | - | SL1555 | putative LysR-family transcriptional | 1.7 | PF03466.13 |
| STM_DT2_31051 | rpoD | SL3185 | RNA polymerase sigma-70 factor | 1.7 | PF04545.9 |
| STM_DT2_32211 | - | SL3301 | conserved hypothetical protein | 1.7 | PF04055.14 |
| STM_DT2_37351 | pstA | SL3822 | phosphate transport system permease protein | 1.7 | PF00528.15 |
| STM_DT2_41611 | - | SL4247 | putative membrane protein | 1.7 | PF06977.4 |
| STM_DT2_06451 | - | SL0642 | conserved hypothetical protein | 1.7 | PF08238.5 |
| STM_DT2_18461 | cheB | SL1852 | Chemotaxis response regulator protein | 1.7 | PF01339.10 |
| STM_DT2_25811 | - | SL2582 | putative bacteriophage protein | 1.7 | PF07105.4 |
| STM_DT2_15341 | rimL | SL1541 | ribosomal-protein-serine acetyltransferase | 1.6 | PF00583.17 |
| STM_DT2_37281 | yidZ | SL3815 | putative LysR-family transcriptionalregulator | 1.6 | PF03466.13 |
| STM_DT2_13391 | ssaH | SL1341 | Type three secretion system apparatus | 1.6 | PF06287.4 |
| STM_DT2_29041 | mutH | SL2983 | DNA mismatch repair protein mutH | 1.6 | PF02976.8 |
| STM_DT2_17531 | - | SL1762 | putative membrane protein | 1.5 | PF06173.5 |
| STM_DT2_22871 | nuoL | SL2287 | NADH dehydrogenase I chain L | 1.5 | PF00662.13 |
| STM_DT2_28261 | pcm | SL2905 | Protein-L-isoaspartate O-methyltransferase | 1.5 | PF01135.12 |
| STM_DT2_31481 | - | SL3228 | PTS system fructose-specific EIIbc component | 1.5 | PF02378.11 |
| STM_DT2_36581 | - | SL3740 | putative glycosyl hydrolase | 1.5 | PF00232.11 |
| STM_DT2_22581 | - | SL2258 | putative 2,4-dihydroxyhept-2-ene-1,7-dioic acid aldolase | 1.4 | PF03328.7 |
| STM_DT2_39351 | ydeV | SL4021 | putative sugar kinase | 1.4 | PF00370.14 |
| STM_DT2_40071 | rpoC | SL4093 | DNA-directed RNA polymerase, beta'-subunit | 1.3 | PF04997.5 |
| STM_DT2_07811 | moaC | SL0780 | molybdenum cofactor biosynthesis prot | 1.2 | PF01967.14 |
| STM_DT2_19741 | - | SL1977 | conserved hypothetical protein | 1.2 | PF06167.5 |
| STM_DT2_37021 | torC | SL3789 | cytochrome c-type protein2.7.1.58) | 1.2 | PF03264.7 |
| STM_DT2_35471 | bax | SL3628 | putative exported amidase | 1.2 | PF01832.13 |
| STM_DT2_00361 | - | SL0036 | putative secreted sulfatase | 1.2 | PF00884.16 |
| STM_DT2_06181 | dcuC | SL0615 | C4-dicarboxylate anaerobic carrier | 1.2 | PF03606.8 |
| STM_DT2_31051 | rpoD | SL3185 | RNA polymerase sigma-70 factor | 1.2 | PF04546.6 |
| STM_DT2_38161 | yigA | SL3902 | conserved hypothetical protein | 1.2 | PF04340.5 |
| STM_DT2_15461 | - | SL1553 | putative esterase | 1.1 | PF00135.21 |
| STM_DT2_04411 | ampG | SL0438 | AmpG protein | 1.1 | PF07690.9 |
| STM_DT2_24211 | eutB | SL2421 | ethanolamine ammonia-lyase heavy chain | 1.1 | PF06751.4 |
| STM_DT2_04441 | tig | SL0441 | trigger factor | 1 | PF05698.7 |
| STM_DT2_25911 | rseA | SL2603 | sigma-E factor negative regulatory protein | 1 | PF03872.6 |
| STM_DT2_31731 | - | SL3253 | ATP-dependent RNA helicase (dead-boxprotein) | 1 | PF00270.22 |
| STM_DT2_38661 | glnG | SL3952 | Two-component system, response regulator | 1 | PF02954.12 |
| STM_DT2_42511 | cpdB | SL4336 | 2',3'-cyclic-nucleotide 2'-phosphodiesterase | 1 | PF00149.21 |
| STM_DT2_14661 | - | SL1469 | putative secreted hydrolase | 0.9 | PF02275.11 |
| STM_DT2_15851 | - | SL1592 | conserved hypothetical protein | 0.9 | PF00582.19 |
| STM_DT2_44321 | creB | SL4515 | putative two-component response regulator | 0.9 |  |
| STM_DT2_07971 | rhlE | SL0796 | putative ATP-dependent RNA helicase rhlE | 0.9 | PF00270.22 |
| STM_DT2_38271 | rhtC | SL3913 | threonine efflux protein | 0.9 | PF01810.11 |
| STM_DT2_00871 | yabF | SL0086 | putative NAD(P)H oxidoreductase | 0.8 | PF02525.10 |
| STM_DT2_14721 | - | SL1475 | putative monooxygenase | 0.8 | PF01494.12 |
| STM_DT2_44061 | osmY | SL4489 | Putative periplasmic protein | 0.8 | PF04972.10 |
| STM_DT2_08421 | mdfA | SL0842 | multidrug translocase MdfA | 0.8 | PF07690.9 |
| STM_DT2_07691 | hutH | SL0768 | 6 histidine ammonia-lyase | 0.7 | PF00221.12 |
| STM_DT2_06931 | - | SL0691 | PTR2-family transport protein | 0.7 | PF00854.14 |
| STM_DT2_42361 | sgaE | SL4321 | probable class II aldolase | 0.7 | PF00596.14 |
| STM_DT2_03131 | prfH | SL0311 | putative peptide chain release factor | 0.7 | PF00472.13 |
| STM_DT2_07961 | ybiH | SL0795 | hypothetical tetR-family transcriptio | 0.7 | PF09209.4 |
| STM_DT2_08361 | - | SL0836 | putative inner membrane protein | 0.7 | PF03600.9 |
| STM_DT2_26801 | iroC | SL2759 | putative ABC transporter protein | 0.7 | PF00664.16 |
| STM_DT2_34431 | ugpB | SL3523 | glycerol-3-phosphate-binding periplasmic protein | 0.7 | PF01547.18 |
| STM_DT2_08831 | clpA | SL0884 | ATP-dependent Clp protease ATP-binding subunit ClpA | 0.6 | PF00004.22 |
| STM_DT2_25201 | cadA | SL2521 | lysine decarboxylase | 0.6 | PF03709.8 |
| STM_DT2_27741 | prgH | SL2854 | pathogenicity 1 island effector protein | 0.6 | PF09480.3 |
| STM_DT2_04881 | ybaL | SL0485 | putative transport protein | 0.5 | PF00999.14 |
| STM_DT2_05371 | folD | SL0535 | FolD bifunctional protein | 0.5 | PF02882.12 |
| STM_DT2_08651 | artI | SL0866 | arginine-binding periplasmic protein | 0.5 | PF00497.13 |
| STM_DT2_22531 | glpA | SL2253 | anaerobic glycerol-3-phosphate dehydrogenase subunit A | 0.5 | PF01266.17 |
| STM_DT2_23801 | yfeD | SL2380 | conserved hypothetical protein | 0.5 | PF07037.4 |
| STM_DT2_38031 | hemX | SL3891 | uroporphyrinogen III methylase | 0.5 | PF04375.7 |
| STM_DT2_04641 | ylaB | SL0461 | hypothetical 58.9 kDa protein in tesb | 0.4 | PF00563.13 |
| STM_DT2_08241 | - | SL0824 | hypothetical ABC transporter ATP-binding protein | 0.4 | PF00005.20 |
| STM_DT2_09841 | - | SL0987 | putative Host Specificity Protein | 0.4 | PF12421.1 |
| STM_DT2_12631 | - | SL1265 | Putative DNA/RNA non-specific endonuclease | 0.4 | PF01223.16 |
| STM_DT2_29791 | - | SL3059 | putative oxidoreductase | 0.4 | PF08125.6 |
| STM_DT2_24721 | guaB | SL2472 | inosine-5'-monophosphate dehydrogenase | 0.4 | PF00571.21 |
| STM_DT2_43181 | - | SL4402 | exported protein | 0.4 | PF12276.1 |
| STM_DT2_40341 | aceK | SL4120 | isocitrate dehydrogenase kinase/phosphatase | 0.4 | PF06315.4 |
| STM_DT2_04471 | lon | SL0444 | Lon protease | 0.3 | PF05362.6 |
| STM_DT2_04731 | acrR | SL0470 | potential acrAB operon repressor | 0.3 | PF08361.4 |
| STM_DT2_33851 | - | SL3465 | heat shock protein | 0.3 | PF01430.12 |
| STM_DT2_11711 | - | SL1173 | putative mutT family protein | 0.3 | PF00293.21 |
| STM_DT2_31061 | mug | SL3186 | G/U mismatch-specific DNA glycosylase | 0.3 | PF03167.12 |
| STM_DT2_22231 | - | SL2223 | cytochrome C-type biogenesis proteinH2 | 0.2 | PF03918.7 |
| STM_DT2_16861 | narK | SL1693 | nitrite extrusion protein (nitrite facilitator) | 0.2 | PF07690.9 |
| STM_DT2_23721 | - | SL2372 | putative membrane protein | 0.2 | PF00654.13 |
| STM_DT2_37241 | intA | SL3811 | putative phage integrase (pseudogene) | 0.2 | PF00589.15 |
| STM_DT2_16201 | - | SL1627 | conserved hypothetical protein | 0.2 | PF00563.13 |
| STM_DT2_23411 | - | SL2341 | putative transmembrane transporter | 0.2 | PF07690.9 |
| STM_DT2_07231 | cydA | SL0722 | cytochrome d ubiquinol oxidase subuni | 0.1 | PF01654.10 |
| STM_DT2_35761 | lctP | SL3657 | L-lactate permease | 0.1 | PF02652.7 |
| STM_DT2_09311 | ycbB | SL0932 | putative exported protein | 0.1 | PF03734.7 |
| STM_DT2_18871 | amyA | SL1892 | cytoplasmic alpha-amylase | 0.1 | PF00128.17 |
| STM_DT2_23301 | - | SL2330 | putative transcriptional regulator | 0.1 | PF08448.3 |
| STM_DT2_32081 | - | SL3288 | conserved hypothetical protein | 0.1 | PF08282.5 |
| STM_DT2_16211 | - | SL1628 | hypothetical protein | 0.1 | PF00563.13 |
| STM_DT2_20761 | wzx | SL2079 | putative transmembrane transport protein | 0.1 | PF01943.10 |
| STM_DT2_21561 | - | SL2156 | putative n-hydroxybenzoate transporter | 0.1 | PF07690.9 |
| STM_DT2_32651 | mreC | SL3345 | rod shape-determining protein | 0.1 | PF04085.7 |
| STM_DT2_44091 | - | SL4492 | conserved hypothetical protein | 0.1 | PF04055.14 |
| STM_DT2_13101 | pykF | SL1312 | pyruvate kinase | 0.1 | PF00224.14 |
| STM_DT2_14261 | dmsA1 | SL1429 | putative dimethyl sulphoxide reductase subunit | 0.1 | PF00384.15 |
| STM_DT2_00221 | bcfB | SL0022 | fimbrial chaperone | 0 | PF02753.10 |
| STM_DT2_00241 | bcfD | SL0024 | fimbrial subunit | 0 | PF00419.13 |
| STM_DT2_00271 | bcfG | SL0027 | fimbrial chaperone | 0 | PF00345.13 |
| STM_DT2_00471 | ileS | SL0047 | isoleucyl-tRNA synthetase | 0 | PF06827.7 |
| STM_DT2_00471 | ileS | SL0047 | isoleucyl-tRNA synthetase | 0 | PF08264.6 |
| STM_DT2_00541 | citA | SL0054 | 9 sensor kinase cita | 0 | PF02518.19 |
| STM_DT2_00561 | - | SL0056 | putative glutaconyl-CoA decarboxylase | 0 | PF00364.15 |
| STM_DT2_00561 | - | SL0056 | putative glutaconyl-CoA decarboxylase | 0 | PF00682.12 |
| STM_DT2_01591 | - | SL0157 | putative exported protein | 0 | PF11737.1 |
| STM_DT2_01901 | ligT | SL0189 | 2'-5' RNA ligase | 0 | PF02834.9 |
| STM_DT2_02141 | - | SL0213 | putative inner membrane protein | 0 | PF00083.17 |
| STM_DT2_02331 | dnaE | SL0232 | DNA polymerase III, alpha chain | 0 | PF01336.18 |
| STM_DT2_02331 | dnaE | SL0232 | DNA polymerase III, alpha chain | 0 | PF02811.12 |
| STM_DT2_02331 | dnaE | SL0232 | DNA polymerase III, alpha chain | 0 | PF07733.5 |
| STM_DT2_02551 | yafE | SL0254 | putative methyltransferase | 0 | PF08241.5 |
| STM_DT2_02811 | sciS | SL0280 | probable IcmF-related integral membrane ATP-binding protein/virulence associated protein | 0 | PF06744.5 |
| STM_DT2_03211 | - | SL0319 | putative IS3 transposase | 0 | PF00665.19 |
| STM_DT2_03691 | - | SL0368 | puative autotransporter/virulence factor | 0 | PF03797.12 |
| STM_DT2_03891 | yajF | SL0388 | hypothetical ROK-family protein | 0 | PF00480.13 |
| STM_DT2_03971 | malZ | SL0396 | maltodextrin glucosidase | 0 | PF00128.17 |
| STM_DT2_03971 | malZ | SL0396 | maltodextrin glucosidase | 0 | PF02568.7 |
| STM_DT2_04211 | - | SL0419 | thiamine biosynthesis protein ThiI | 0 | PF02926.10 |
| STM_DT2_04441 | tig | SL0441 | trigger factor | 0 | PF00254.21 |
| STM_DT2_04441 | tig | SL0441 | trigger factor | 0 | PF05697.6 |
| STM_DT2_04471 | lon | SL0444 | Lon protease | 0 | PF00004.22 |
| STM_DT2_04471 | lon | SL0444 | Lon protease | 0 | PF02190.9 |
| STM_DT2_04581 | mdlB | SL0455 | putative ABC transporter ATP-binding | 0 | PF00005.20 |
| STM_DT2_04631 | ybaZ | SL0460 | putative methylated-DNA methyltransferase | 0 | PF01035.13 |
| STM_DT2_04731 | acrR | SL0470 | potential acrAB operon repressor | 0 | PF00440.16 |
| STM_DT2_04851 | hemH | SL0482 | ferrochelatase | 0 | PF00762.12 |
| STM_DT2_04851 | hemH | SL0482 | ferrochelatase | 0 | PF02254.11 |
| STM_DT2_04901 | ushA | SL0487 | UDP-sugar hydrolase | 0 | PF02872.11 |
| STM_DT2_04941 | ybaR | SL0491 | copper-transporting ATPase | 0 | PF00122.13 |
| STM_DT2_04941 | ybaR | SL0491 | copper-transporting ATPase | 0 | PF00403.19 |
| STM_DT2_04941 | ybaR | SL0491 | copper-transporting ATPase | 0 | PF00403.19 |
| STM_DT2_04941 | ybaR | SL0491 | copper-transporting ATPase | 0 | PF00702.19 |
| STM_DT2_05371 | folD | SL0535 | FolD bifunctional protein | 0 | PF00763.16 |
| STM_DT2_05471 | fimW | SL0545 | fimbriae w protein | 0 | PF00196.12 |
| STM_DT2_05621 | manR | SL0559 | similar to lmo0785 protein (sigma 54-associated) | 0 | PF00158.19 |
| STM_DT2_05621 | manR | SL0559 | similar to lmo0785 protein (sigma 54-associated) | 0 | PF00874.13 |
| STM_DT2_05621 | manR | SL0559 | similar to lmo0785 protein (sigma 54-associated) | 0 | PF01380.15 |
| STM_DT2_05761 | fepA | SL0573 | ferrienterobactin receptor precursor | 0 | PF07715.8 |
| STM_DT2_05791 | entF | SL0576 | enterobactin synthetase component F | 0 | PF00550.18 |
| STM_DT2_05791 | entF | SL0576 | enterobactin synthetase component F | 0 | PF00668.13 |
| STM_DT2_05791 | entF | SL0576 | enterobactin synthetase component F | 0 | PF00975.13 |
| STM_DT2_05911 | cstA | SL0588 | carbon starvation protein A | 0 | PF02554.7 |
| STM_DT2_06121 | citF | SL0609 | citrate lyase alpha chain | 0 | PF04223.5 |
| STM_DT2_06321 | ybeA | SL0629 | conserved hypothetical protein | 0 | PF08238.5 |
| STM_DT2_06321 | ybeA | SL0629 | conserved hypothetical protein | 0 | PF08238.5 |
| STM_DT2_06321 | ybeA | SL0629 | conserved hypothetical protein | 0 | PF08238.5 |
| STM_DT2_06321 | ybeA | SL0629 | conserved hypothetical protein | 0 | PF08238.5 |
| STM_DT2_06321 | ybeA | SL0629 | conserved hypothetical protein | 0 | PF08238.5 |
| STM_DT2_06321 | ybeA | SL0629 | conserved hypothetical protein | 0 | PF08238.5 |
| STM_DT2_06321 | ybeA | SL0629 | conserved hypothetical protein | 0 | PF08238.5 |
| STM_DT2_06561 | ybeJ | SL0654 | ABC transporter periplasmic binding protein (glutamate/aspartate?) | 0 | PF00497.13 |
| STM_DT2_06701 | glnS | SL0668 | glutaminyl-tRNA synthetase | 0 | PF00749.14 |
| STM_DT2_06761 | - | SL0674 | lysR-family transcriptional regulator | 0 | PF00126.20 |
| STM_DT2_07061 | - | SL0705 | ABC transporter domain protein | 0 | PF00676.13 |
| STM_DT2_07191 | sucA | SL0718 | 2-oxoglutarate dehydrogenase E1 compo | 0 | PF02779.17 |
| STM_DT2_07211 | sucC | SL0720 | succinyl-CoA synthetase beta chain | 0 | PF00549.12 |
| STM_DT2_07221 | sucD | SL0721 | succinyl-CoA synthetase alpha chain | 0 | PF00549.12 |
| STM_DT2_07221 | sucD | SL0721 | succinyl-CoA synthetase alpha chain | 0 | PF02629.12 |
| STM_DT2_07301 | tolA | SL0729 | tolA protein | 0 | PF06519.4 |
| STM_DT2_07731 | bioF | SL0772 | 8-amino-7-oxononanoate synthase | 0 | PF00155.14 |
| STM_DT2_07891 | ybhO | SL0788 | putative phospholipase | 0 | PF00614.15 |
| STM_DT2_07961 | ybiH | SL0795 | hypothetical tetR-family transcriptio | 0 | PF00440.16 |
| STM_DT2_07991 | ybiB | SL0798 | putative glycosyltransferase | 0 | PF02885.10 |
| STM_DT2_08031 | - | SL0802 | putative membrane protein | 0 | PF00924.11 |
| STM_DT2_08141 | ybiT | SL0813 | ABC transporter ATP-binding protein | 0 | PF00005.20 |
| STM_DT2_08141 | ybiT | SL0813 | ABC transporter ATP-binding protein | 0 | PF00005.20 |
| STM_DT2_08141 | ybiT | SL0813 | ABC transporter ATP-binding protein | 0 | PF00005.20 |
| STM_DT2_08141 | ybiT | SL0813 | ABC transporter ATP-binding protein | 0 | PF08352.5 |
| STM_DT2_08141 | ybiT | SL0813 | ABC transporter ATP-binding protein | 0 | PF08352.5 |
| STM_DT2_08661 | artP | SL0867 | arginine transport ATP-binding protein ArtP | 0 | PF00005.20 |
| STM_DT2_08661 | artP | SL0867 | arginine transport ATP-binding protein ArtP | 0 | PF02861.13 |
| STM_DT2_08661 | artP | SL0867 | arginine transport ATP-binding protein ArtP | 0 | PF07724.7 |
| STM_DT2_08661 | artP | SL0867 | arginine transport ATP-binding protein ArtP | 0 | PF10431.2 |
| STM_DT2_08971 | ftsK | SL0898 | cell division protein FtsK | 0 | PF01580.11 |
| STM_DT2_08971 | ftsK | SL0898 | cell division protein FtsK | 0 | PF09397.3 |
| STM_DT2_09311 | ycbB | SL0932 | putative exported protein | 0 | PF01471.11 |
| STM_DT2_09441 | recE | SL0946 | exodeoxyribonuclease viii | 0 | PF06630.4 |
| STM_DT2_09681 | - | SL0971 | Putative Prophage Protein | 0 | PF05136.6 |
| STM_DT2_09741 | - | SL0977 | Tail Protein | 0 | PF02368.11 |
| STM_DT2_09841 | - | SL0987 | putative Host Specificity Protein | 0 | PF09327.4 |
| STM_DT2_09971 | - | SL1000 | conserved hypothetical protein | 0 | PF00111.20 |
| STM_DT2_09971 | - | SL1000 | conserved hypothetical protein | 0 | PF03473.10 |
| STM_DT2_10001 | pqiA | SL1003 | putative inner membrane protein | 0 | PF04403.6 |
| STM_DT2_10191 | - | SL1022 | putative transcriptional regulator | 0 | PF00165.16 |
| STM_DT2_10351 | hpaB | SL1038 | 4-hydroxyphenylacetate 3-monooxygenas | 0 | PF11794.1 |
| STM_DT2_10851 | mdoH | SL1088 | Glucans biosynthesis glucosyltransferase H | 0 | PF00535.19 |
| STM_DT2_11191 | ams | SL1122 | ribonuclease E | 0 | PF00575.16 |
| STM_DT2_11191 | ams | SL1122 | ribonuclease E | 0 | PF10150.2 |
| STM_DT2_11191 | ams | SL1122 | ribonuclease E | 0 | PF12111.1 |
| STM_DT2_11481 | - | SL1150 | putative TetR-family regulatory protein | 0 | PF00440.16 |
| STM_DT2_11511 | mfd | SL1153 | transcription-repair coupling factor | 0 | PF00271.24 |
| STM_DT2_11511 | mfd | SL1153 | transcription-repair coupling factor | 0 | PF02559.9 |
| STM_DT2_11511 | mfd | SL1153 | transcription-repair coupling factor | 0 | PF03461.8 |
| STM_DT2_11921 | - | SL1195 | putative ABC transport ATP-binding subunit | 0 | PF00005.20 |
| STM_DT2_11941 | - | SL1197 | conserved hypothetical protein | 0 | PF03860.9 |
| STM_DT2_11941 | - | SL1197 | conserved hypothetical protein | 0 | PF03860.9 |
| STM_DT2_12711 | pheT | SL1272 | Phenylalanyl-tRNA synthetase beta cha | 0 | PF01588.13 |
| STM_DT2_12711 | pheT | SL1272 | Phenylalanyl-tRNA synthetase beta cha | 0 | PF03147.7 |
| STM_DT2_12711 | pheT | SL1272 | Phenylalanyl-tRNA synthetase beta cha | 0 | PF03483.10 |
| STM_DT2_12711 | pheT | SL1272 | Phenylalanyl-tRNA synthetase beta cha | 0 | PF03484.8 |
| STM_DT2_12751 | btuD | SL1276 | vitamin B12 ABC transport ATP-binding | 0 | PF00005.20 |
| STM_DT2_12771 | - | SL1278 | conserved hypothetical protein | 0 | PF00563.13 |
| STM_DT2_12821 | pps | SL1283 | phosphoenolpyruvate synthase | 0 | PF00391.16 |
| STM_DT2_12821 | pps | SL1283 | phosphoenolpyruvate synthase | 0 | PF02896.11 |
| STM_DT2_12891 | - | SL1290 | Putative acyl-CoA dehydrogenase | 0 | PF00441.17 |
| STM_DT2_12891 | - | SL1290 | Putative acyl-CoA dehydrogenase | 0 | PF02770.12 |
| STM_DT2_12941 | - | SL1296 | putative MFS-family transport protein | 0 | PF07690.9 |
| STM_DT2_13001 | - | SL1302 | putative transporter | 0 | PF00375.11 |
| STM_DT2_13001 | - | SL1302 | putative transporter | 0 | PF02887.9 |
| STM_DT2_13151 | ttrA | SL1317 | tetrathionate reductase subunit A | 0 | PF00384.15 |
| STM_DT2_13151 | ttrA | SL1317 | tetrathionate reductase subunit A | 0 | PF01568.14 |
| STM_DT2_13151 | ttrA | SL1317 | tetrathionate reductase subunit A | 0 | PF04879.9 |
| STM_DT2_13501 | ssaQ | SL1352 | putative type III secretion protein | 0 | PF01052.13 |
| STM_DT2_13831 | nth | SL1385 | endonuclease III | 0 | PF00633.16 |
| STM_DT2_13831 | nth | SL1385 | endonuclease III | 0 | PF10576.2 |
| STM_DT2_13991 | rstB | SL1402 | two component sensor kinase | 0 | PF00512.18 |
| STM_DT2_13991 | rstB | SL1402 | two component sensor kinase | 0 | PF00672.18 |
| STM_DT2_14061 | pntA | SL1409 | pyridine nucleotide transhydrogenase | 0 | PF01262.14 |
| STM_DT2_14061 | pntA | SL1409 | pyridine nucleotide transhydrogenase | 0 | PF05222.8 |
| STM_DT2_14151 | - | SL1418 | putative regulatory protein | 0 | PF00480.13 |
| STM_DT2_14211 | - | SL1424 | putative ABC transporter membrane protein | 0 | PF01568.14 |
| STM_DT2_14211 | - | SL1424 | putative ABC transporter membrane protein | 0 | PF04879.9 |
| STM_DT2_14431 | - | SL1446 | putative membrane protein | 0 | PF07690.9 |
| STM_DT2_14921 | fdnH | SL1499 | formate dehydrogenase, nitrate-inducible, iron-sulfur subunit | 0 | PF09163.4 |
| STM_DT2_15001 | narZ | SL1508 | respiratory nitrate reductase 2 alpha chain | 0 | PF00384.15 |
| STM_DT2_15001 | narZ | SL1508 | respiratory nitrate reductase 2 alpha chain | 0 | PF01568.14 |
| STM_DT2_15121 | - | SL1520 | putative NADP-dependent oxidoreductase | 0 | PF00107.19 |
| STM_DT2_15481 | - | SL1555 | putative LysR-family transcriptional | 0 | PF00126.20 |
| STM_DT2_15501 | adhC | SL1557 | alcohol dehydrogenase class III (ec 1.1.1.1) (ec 1.2.1.1) (fdh) (faldh) | 0 | PF00107.19 |
| STM_DT2_15501 | adhC | SL1557 | alcohol dehydrogenase class III (ec 1.1.1.1) (ec 1.2.1.1) (fdh) (faldh) | 0 | PF08240.5 |
| STM_DT2_15741 | - | SL1581 | probable pyruvate-flavodoxin oxidoreductase | 0 | PF01855.12 |
| STM_DT2_15741 | - | SL1581 | probable pyruvate-flavodoxin oxidoreductase | 0 | PF02775.14 |
| STM_DT2_15741 | - | SL1581 | probable pyruvate-flavodoxin oxidoreductase | 0 | PF10371.2 |
| STM_DT2_15741 | - | SL1581 | probable pyruvate-flavodoxin oxidoreductase | 0 | PF00582.19 |
| STM_DT2_16051 | tpx | SL1612 | Probable thiol peroxidase | 0 | PF08534.3 |
| STM_DT2_16071 | - | SL1614 | putative membrane protein | 0 | PF05128.5 |
| STM_DT2_16271 | - | SL1634 | conserved hypothetical protein | 0 | PF00989.17 |
| STM_DT2_16271 | - | SL1634 | conserved hypothetical protein | 0 | PF00990.14 |
| STM_DT2_16871 | narX | SL1694 | nitrate/nitrite sensor protein NarX | 0 | PF00672.18 |
| STM_DT2_16871 | narX | SL1694 | nitrate/nitrite sensor protein NarX | 0 | PF02518.19 |
| STM_DT2_16871 | narX | SL1694 | nitrate/nitrite sensor protein NarX | 0 | PF07730.6 |
| STM_DT2_17081 | hyaC | SL1716 | probable Ni/Fe-hydrogenase 1 b-type cytochrome subunit | 0 | PF00033.12 |
| STM_DT2_17081 | hyaC | SL1716 | probable Ni/Fe-hydrogenase 1 b-type cytochrome subunit | 0 | PF03471.10 |
| STM_DT2_17081 | hyaC | SL1716 | probable Ni/Fe-hydrogenase 1 b-type cytochrome subunit | 0 | PF03741.9 |
| STM_DT2_17751 | sopE2 | SL1784 | invasion-associated secreted effector protein (sopE2) | 0 | PF05364.7 |
| STM_DT2_17751 | sopE2 | SL1784 | invasion-associated secreted effector protein (sopE2) | 0 | PF07487.6 |
| STM_DT2_18381 | argS | SL1844 | Arginyl-tRNA synthetase | 0 | PF03485.9 |
| STM_DT2_18381 | argS | SL1844 | Arginyl-tRNA synthetase | 0 | PF05746.8 |
| STM_DT2_18461 | cheB | SL1852 | Chemotaxis response regulator protein | 0 | PF00072.17 |
| STM_DT2_18461 | cheB | SL1852 | Chemotaxis response regulator protein | 0 | PF00672.18 |
| STM_DT2_19061 | rcsA | SL1911 | colanic acid capsullar biosynthesis activation protein A | 0 | PF00196.12 |
| STM_DT2_19451 | - | SL1948 | putative peptidase | 0 | PF04586.10 |
| STM_DT2_19601 | - | SL1963 | putative prophage protein | 0 | PF06069.4 |
| STM_DT2_19731 | - | SL1976 | phage integrase | 0 | PF00589.15 |
| STM_DT2_20101 | - | SL2013 | putative propanediol diffusion facilitator | 0 | PF00230.13 |
| STM_DT2_20191 | pduK | SL2022 | propanediol utilization protein | 0 | PF00936.12 |
| STM_DT2_20311 | pduX | SL2034 | putative propanediol utilization protein | 0 | PF00288.19 |
| STM_DT2_20451 | hisG | SL2048 | ATP phosphoribosyltransferase | 0 | PF08029.4 |
| STM_DT2_20721 | galF | SL2075 | UTP-glucose-1-phosphate uridylyltransferase | 0 | PF00483.16 |
| STM_DT2_20791 | cpsB | SL2082 | mannose-1-phosphate guanylyltransferase | 0 | PF01050.11 |
| STM_DT2_20901 | wzc | SL2093 | tyrosine-protein kinase | 0 | PF01656.16 |
| STM_DT2_20901 | wzc | SL2093 | tyrosine-protein kinase | 0 | PF02706.8 |
| STM_DT2_21391 | yehY | SL2142 | putative permease transmembrane component | 0 | PF00528.15 |
| STM_DT2_21661 | mglA | SL2166 | galactoside transport atp-binding protein mgla | 0 | PF00005.20 |
| STM_DT2_21661 | mglA | SL2166 | galactoside transport atp-binding protein mgla | 0 | PF00005.20 |
| STM_DT2_21771 | lysP | SL2177 | lysine-specific permease | 0 | PF00324.14 |
| STM_DT2_22401 | rcsC | SL2240 | sensor protein RcsC | 0 | PF00072.17 |
| STM_DT2_22401 | rcsC | SL2240 | sensor protein RcsC | 0 | PF00512.18 |
| STM_DT2_22401 | rcsC | SL2240 | sensor protein RcsC | 0 | PF02518.19 |
| STM_DT2_22401 | rcsC | SL2240 | sensor protein RcsC | 0 | PF09456.3 |
| STM_DT2_22401 | rcsC | SL2240 | sensor protein RcsC | 0 | PF04324.8 |
| STM_DT2_22871 | nuoL | SL2287 | NADH dehydrogenase I chain L | 0 | PF00361.13 |
| STM_DT2_22991 | lrhA | SL2299 | NADH dehydrogenase operon transcriptional regulator | 0 | PF00126.20 |
| STM_DT2_22991 | lrhA | SL2299 | NADH dehydrogenase operon transcriptional regulator | 0 | PF03466.13 |
| STM_DT2_23171 | - | SL2317 | putative glutathione-S transferase | 0 | PF02798.13 |
| STM_DT2_23301 | - | SL2330 | putative transcriptional regulator | 0 | PF00158.19 |
| STM_DT2_23301 | - | SL2330 | putative transcriptional regulator | 0 | PF02954.12 |
| STM_DT2_23731 | - | SL2373 | putative decarboxylase | 0 | PF02775.14 |
| STM_DT2_23731 | - | SL2373 | putative decarboxylase | 0 | PF02776.11 |
| STM_DT2_23741 | - | SL2374 | putative ion-channel protein | 0 | PF00248.14 |
| STM_DT2_23771 | nupC | SL2377 | nucleoside permease NupC | 0 | PF01773.13 |
| STM_DT2_23771 | nupC | SL2377 | nucleoside permease NupC | 0 | PF07662.6 |
| STM_DT2_23931 | cysK | SL2393 | cysteine synthase A | 0 | PF00291.18 |
| STM_DT2_24041 | cysA | SL2404 | sulphate transport ATP-binding protein CysA | 0 | PF00005.20 |
| STM_DT2_24711 | guaA | SL2471 | GMP synthase [glutamine-hydrolyzing] | 0 | PF00117.21 |
| STM_DT2_24711 | guaA | SL2471 | GMP synthase [glutamine-hydrolyzing] | 0 | PF00958.15 |
| STM_DT2_24711 | guaA | SL2471 | GMP synthase [glutamine-hydrolyzing] | 0 | PF02540.10 |
| STM_DT2_24721 | guaB | SL2472 | inosine-5'-monophosphate dehydrogenase | 0 | PF00571.21 |
| STM_DT2_24741 | shdA | SL2474 | host colonisation factor (ShdA) | 0 | PF03797.12 |
| STM_DT2_24751 | ratB | SL2475 | putative outer membrane protein (RatB) (pseudogene) | 0 | PF05688.4 |
| STM_DT2_24751 | ratB | SL2475 | putative outer membrane protein (RatB) (pseudogene) | 0 | PF05688.4 |
| STM_DT2_24751 | ratB | SL2475 | putative outer membrane protein (RatB) (pseudogene) | 0 | PF05688.4 |
| STM_DT2_24751 | ratB | SL2475 | putative outer membrane protein (RatB) (pseudogene) | 0 | PF05688.4 |
| STM_DT2_24751 | ratB | SL2475 | putative outer membrane protein (RatB) (pseudogene) | 0 | PF05688.4 |
| STM_DT2_24751 | ratB | SL2475 | putative outer membrane protein (RatB) (pseudogene) | 0 | PF05688.4 |
| STM_DT2_24751 | ratB | SL2475 | putative outer membrane protein (RatB) (pseudogene) | 0 | PF05688.4 |
| STM_DT2_24751 | ratB | SL2475 | putative outer membrane protein (RatB) (pseudogene) | 0 | PF05688.4 |
| STM_DT2_24751 | ratB | SL2475 | putative outer membrane protein (RatB) (pseudogene) | 0 | PF05689.4 |
| STM_DT2_24751 | ratB | SL2475 | putative outer membrane protein (RatB) (pseudogene) | 0 | PF05689.4 |
| STM_DT2_24751 | ratB | SL2475 | putative outer membrane protein (RatB) (pseudogene) | 0 | PF05689.4 |
| STM_DT2_24751 | ratB | SL2475 | putative outer membrane protein (RatB) (pseudogene) | 0 | PF05689.4 |
| STM_DT2_24751 | ratB | SL2475 | putative outer membrane protein (RatB) (pseudogene) | 0 | PF05689.4 |
| STM_DT2_24751 | ratB | SL2475 | putative outer membrane protein (RatB) (pseudogene) | 0 | PF05689.4 |
| STM_DT2_24751 | ratB | SL2475 | putative outer membrane protein (RatB) (pseudogene) | 0 | PF05689.4 |
| STM_DT2_25011 | hscB | SL2502 | Co-chaperone protein hscB | 0 | PF00226.24 |
| STM_DT2_25171 | hmpA | SL2518 | flavohemoprotein (haemoglobin-like protein) | 0 | PF00175.14 |
| STM_DT2_25191 | cadB | SL2520 | probable cadaverine/lysine antiporter | 0 | PF00970.17 |
| STM_DT2_25201 | cadA | SL2521 | lysine decarboxylase | 0 | PF01276.13 |
| STM_DT2_25201 | cadA | SL2521 | lysine decarboxylase | 0 | PF03711.8 |
| STM_DT2_25251 | - | SL2526 | putative sensor kinase protein | 0 | PF00512.18 |
| STM_DT2_25251 | - | SL2526 | putative sensor kinase protein | 0 | PF00672.18 |
| STM_DT2_25251 | - | SL2526 | putative sensor kinase protein | 0 | PF02518.19 |
| STM_DT2_25511 | - | SL2552 | similar to a DNA recombinase | 0 | PF07484.5 |
| STM_DT2_25571 | - | SL2558 | putative prophage protein | 0 | PF06791.6 |
| STM_DT2_25571 | - | SL2558 | putative prophage protein | 0 | PF09718.3 |
| STM_DT2_25811 | - | SL2582 | putative bacteriophage protein | 0 | PF03873.6 |
| STM_DT2_26031 | - | SL2615 | putative acyl-CoA synthetase | 0 | PF00583.17 |
| STM_DT2_26311 | recN | SL2656 | DNA repair protein | 0 | PF02463.12 |
| STM_DT2_26801 | iroC | SL2759 | putative ABC transporter protein | 0 | PF00005.20 |
| STM_DT2_26801 | iroC | SL2759 | putative ABC transporter protein | 0 | PF00005.20 |
| STM_DT2_26801 | iroC | SL2759 | putative ABC transporter protein | 0 | PF00664.16 |
| STM_DT2_27131 | nrdE | SL2792 | ribonucleoside-diphosphate reductase2 alpha chain | 0 | PF00317.14 |
| STM_DT2_27131 | nrdE | SL2792 | ribonucleoside-diphosphate reductase2 alpha chain | 0 | PF08343.3 |
| STM_DT2_27161 | proW | SL2795 | glycine betaine/L-proline transport system permease protein P | 0 | PF00528.15 |
| STM_DT2_28091 | mutS | SL2888 | DNA mismatch repair protein | 0 | PF00488.14 |
| STM_DT2_28091 | mutS | SL2888 | DNA mismatch repair protein | 0 | PF01624.13 |
| STM_DT2_28091 | mutS | SL2888 | DNA mismatch repair protein | 0 | PF05188.10 |
| STM_DT2_28091 | mutS | SL2888 | DNA mismatch repair protein | 0 | PF05190.11 |
| STM_DT2_28271 | surE | SL2906 | Acid phosphatase surE | 0 | PF01975.10 |
| STM_DT2_28271 | surE | SL2906 | Acid phosphatase surE | 0 | PF05958.4 |
| STM_DT2_29021 | ptsP | SL2981 | phosphoenolpyruvate-protein phosphotransferase | 0 | PF00391.16 |
| STM_DT2_29021 | ptsP | SL2981 | phosphoenolpyruvate-protein phosphotransferase | 0 | PF01590.19 |
| STM_DT2_29021 | ptsP | SL2981 | phosphoenolpyruvate-protein phosphotransferase | 0 | PF05524.6 |
| STM_DT2_29081 | ygeD | SL2987 | putative membrane protein | 0 | PF07690.9 |
| STM_DT2_29251 | - | SL3005 | hypothetical protein | 0 | PF00345.13 |
| STM_DT2_29791 | - | SL3059 | putative oxidoreductase | 0 | PF01232.16 |
| STM_DT2_29791 | - | SL3059 | putative oxidoreductase | 0 | PF03709.8 |
| STM_DT2_29791 | - | SL3059 | putative oxidoreductase | 0 | PF03711.8 |
| STM_DT2_30091 | speC | SL3089 | ornithine decarboxylase, constitutive(ec 4.1.1.17) | 0 | PF03328.7 |
| STM_DT2_30141 | citE | SL3094 | possible citrate lyase | 0 | PF00196.12 |
| STM_DT2_30201 | - | SL3100 | possible amino acid transport protein | 0 | PF01058.15 |
| STM_DT2_30441 | hyb0 | SL3124 | hydrogenase-2 small chain protein | 0 | PF00521.13 |
| STM_DT2_30441 | hyb0 | SL3124 | hydrogenase-2 small chain protein | 0 | PF03989.6 |
| STM_DT2_30441 | hyb0 | SL3124 | hydrogenase-2 small chain protein | 0 | PF03989.6 |
| STM_DT2_31051 | rpoD | SL3185 | RNA polymerase sigma-70 factor | 0 | PF00140.13 |
| STM_DT2_31051 | rpoD | SL3185 | RNA polymerase sigma-70 factor | 0 | PF03979.7 |
| STM_DT2_31051 | rpoD | SL3185 | RNA polymerase sigma-70 factor | 0 | PF04539.9 |
| STM_DT2_31051 | rpoD | SL3185 | RNA polymerase sigma-70 factor | 0 | PF04542.7 |
| STM_DT2_31091 | cheM | SL3189 | methyl-accepting chemotaxis protein II | 0 | PF00015.14 |
| STM_DT2_31091 | cheM | SL3189 | methyl-accepting chemotaxis protein II | 0 | PF00672.18 |
| STM_DT2_31111 | - | SL3191 | probable aminotransferase | 0 | PF00724.13 |
| STM_DT2_31121 | fadH | SL3192 | 2,4-dienoyl-CoA reductase [NADPH] (ec1.3.1.34) (2,4-dienoyl coenzyme A reductase) | 0 | PF07992.7 |
| STM_DT2_31481 | - | SL3228 | PTS system fructose-specific EIIbc component | 0 | PF02302.10 |
| STM_DT2_31721 | - | SL3252 | probable amino acid permease | 0 | PF03222.6 |
| STM_DT2_31721 | - | SL3252 | probable amino acid permease | 0 | PF00271.24 |
| STM_DT2_31721 | - | SL3252 | probable amino acid permease | 0 | PF03880.8 |
| STM_DT2_31721 | - | SL3252 | probable amino acid permease | 0 | PF12343.1 |
| STM_DT2_32301 | nanT | SL3310 | putative sialic acid transporter | 0 | PF00083.17 |
| STM_DT2_32311 | nanA | SL3311 | N-acetylneuraminate lyase | 0 | PF00701.15 |
| STM_DT2_32441 | oadA2 | SL3324 | oxaloacetate decarboxylase alpha chain | 0 | PF00682.12 |
| STM_DT2_32441 | oadA2 | SL3324 | oxaloacetate decarboxylase alpha chain | 0 | PF02436.11 |
| STM_DT2_32621 | rnG | SL3342 | ribonuclease G | 0 | PF00575.16 |
| STM_DT2_32961 | sapG | SL3376 | potassium transport protein | 0 | PF02080.14 |
| STM_DT2_32961 | sapG | SL3376 | potassium transport protein | 0 | PF02080.14 |
| STM_DT2_32961 | sapG | SL3376 | potassium transport protein | 0 | PF02254.11 |
| STM_DT2_32991 | yhdM | SL3379 | putative Zn(II)-responsive regulator | 0 | PF00376.16 |
| STM_DT2_32991 | yhdM | SL3379 | putative Zn(II)-responsive regulator | 0 | PF09278.4 |
| STM_DT2_33121 | rplF | SL3392 | 50S ribosomal subunit protein L6 | 0 | PF00347.16 |
| STM_DT2_33461 | yheS | SL3426 | probable ABC transporter ATP-bindingprotein | 0 | PF00005.20 |
| STM_DT2_33461 | yheS | SL3426 | probable ABC transporter ATP-bindingprotein | 0 | PF00005.20 |
| STM_DT2_33551 | argD | SL3435 | acetylornithine aminotransferase | 0 | PF00202.14 |
| STM_DT2_33611 | nirB | SL3441 | nitrite reductase large subunit | 0 | PF00070.20 |
| STM_DT2_33611 | nirB | SL3441 | nitrite reductase large subunit | 0 | PF01077.15 |
| STM_DT2_33611 | nirB | SL3441 | nitrite reductase large subunit | 0 | PF03460.10 |
| STM_DT2_33611 | nirB | SL3441 | nitrite reductase large subunit | 0 | PF04324.8 |
| STM_DT2_34201 | - | SL3500 | putative IclR-family transcriptional regulator | 0 | PF01614.11 |
| STM_DT2_34201 | - | SL3500 | putative IclR-family transcriptional regulator | 0 | PF09339.3 |
| STM_DT2_34321 | - | SL3512 | putative acetyltransferase | 0 | PF00583.17 |
| STM_DT2_34801 | - | SL3560 | putative phosphatase | 0 | PF01569.14 |
| STM_DT2_34871 | - | SL3567 | gntR family regulatory protein | 0 | PF00392.14 |
| STM_DT2_34871 | - | SL3567 | gntR family regulatory protein | 0 | PF07702.6 |
| STM_DT2_35011 | yhjL | SL3581 | putative polysaccharide biosynthesisprotein subunit C | 0 | PF05420.4 |
| STM_DT2_35411 | - | SL3622 | putative lipoprotein | 0 | PF00165.16 |
| STM_DT2_35461 | xylR | SL3627 | xylose operon regulatory protein | 0 | PF00165.16 |
| STM_DT2_35581 | lyxK | SL3639 | putative L-xylulose kinase | 0 | PF00370.14 |
| STM_DT2_35581 | lyxK | SL3639 | putative L-xylulose kinase | 0 | PF02782.9 |
| STM_DT2_35671 | selA | SL3648 | L-seryl-tRNA(Ser) selenium transferase | 0 | PF03841.6 |
| STM_DT2_35851 | gpsA | SL3666 | glycerol-3-phosphate dehydrogenase | 0 | PF01210.16 |
| STM_DT2_36161 | dfp | SL3696 | conserved hypothetical protein | 0 | PF02441.12 |
| STM_DT2_36411 | misL | SL3723 | putative autotransported protein (MisL) | 0 | PF03212.7 |
| STM_DT2_36411 | misL | SL3723 | putative autotransported protein (MisL) | 0 | PF03797.12 |
| STM_DT2_36921 | yidE | SL3774 | putative membrane protein | 0 | PF02080.14 |
| STM_DT2_36921 | yidE | SL3774 | putative membrane protein | 0 | PF02080.14 |
| STM_DT2_36921 | yidE | SL3774 | putative membrane protein | 0 | PF06826.5 |
| STM_DT2_36991 | yhjA | SL3786 | probable cytochrome c peroxidase | 0 | PF00034.14 |
| STM_DT2_36991 | yhjA | SL3786 | probable cytochrome c peroxidase | 0 | PF03150.7 |
| STM_DT2_36991 | yhjA | SL3786 | probable cytochrome c peroxidase | 0 | PF01568.14 |
| STM_DT2_37091 | dgoK | SL3796 | 2-dehydro-3-deoxygalactonokinase (ec | 0 | PF05035.5 |
| STM_DT2_37281 | yidZ | SL3815 | putative LysR-family transcriptionalregulator | 0 | PF00126.20 |
| STM_DT2_37391 | - | SL3826 | putative shikimate 5-dehydrogenase | 0 | PF08501.4 |
| STM_DT2_37711 | yifB | SL3860 | Putative competence protein (chelatase) | 0 | PF01078.14 |
| STM_DT2_37881 | rho | SL3876 | transcription termination factor | 0 | PF00006.18 |
| STM_DT2_37881 | rho | SL3876 | transcription termination factor | 0 | PF07497.5 |
| STM_DT2_37881 | rho | SL3876 | transcription termination factor | 0 | PF07498.5 |
| STM_DT2_38661 | glnG | SL3952 | Two-component system, response regulator | 0 | PF00072.17 |
| STM_DT2_38661 | glnG | SL3952 | Two-component system, response regulator | 0 | PF00158.19 |
| STM_DT2_39121 | rhaR | SL3998 | L-rhamnose operon transcriptional activator | 0 | PF00165.16 |
| STM_DT2_39121 | rhaR | SL3998 | L-rhamnose operon transcriptional activator | 0 | PF02311.12 |
| STM_DT2_39211 | cpxA | SL4007 | two-component sensor kinase protein | 0 | PF00512.18 |
| STM_DT2_39211 | cpxA | SL4007 | two-component sensor kinase protein | 0 | PF00672.18 |
| STM_DT2_39211 | cpxA | SL4007 | two-component sensor kinase protein | 0 | PF02782.9 |
| STM_DT2_39411 | yneB | SL4027 | putative aldolase | 0 | PF01791.2 |
| STM_DT2_39411 | yneB | SL4027 | putative aldolase | 0 | PF00970.17 |
| STM_DT2_39891 | udhA | SL4075 | possible pyridine nucleotide-disulphide oxidoreductase | 0 | PF00070.20 |
| STM_DT2_39891 | udhA | SL4075 | possible pyridine nucleotide-disulphide oxidoreductase | 0 | PF02852.15 |
| STM_DT2_39891 | udhA | SL4075 | possible pyridine nucleotide-disulphide oxidoreductase | 0 | PF07992.7 |
| STM_DT2_40071 | rpoC | SL4093 | DNA-directed RNA polymerase, beta'-subunit | 0 | PF00623.13 |
| STM_DT2_40071 | rpoC | SL4093 | DNA-directed RNA polymerase, beta'-subunit | 0 | PF04983.11 |
| STM_DT2_40071 | rpoC | SL4093 | DNA-directed RNA polymerase, beta'-subunit | 0 | PF04998.10 |
| STM_DT2_40071 | rpoC | SL4093 | DNA-directed RNA polymerase, beta'-subunit | 0 | PF05000.10 |
| STM_DT2_40281 | purD | SL4114 | phosphoribosylglycineamide synthetase | 0 | PF01071.12 |
| STM_DT2_40281 | purD | SL4114 | phosphoribosylglycineamide synthetase | 0 | PF02843.9 |
| STM_DT2_41551 | dcuS | SL4241 | two-component sensor kinase | 0 | PF00989.17 |
| STM_DT2_42001 | yjeQ | SL4286 | putative membrane protein | 0 | PF03193.9 |
| STM_DT2_42071 | mutL | SL4293 | DNA mismatch repair protein | 0 | PF01119.12 |
| STM_DT2_42071 | mutL | SL4293 | DNA mismatch repair protein | 0 | PF02518.19 |
| STM_DT2_42071 | mutL | SL4293 | DNA mismatch repair protein | 0 | PF08676.4 |
| STM_DT2_42151 | rnr | SL4301 | ribonuclease R (RNase R) | 0 | PF00575.16 |
| STM_DT2_42151 | rnr | SL4301 | ribonuclease R (RNase R) | 0 | PF08206.4 |
| STM_DT2_42151 | rnr | SL4301 | ribonuclease R (RNase R) | 0 | PF08206.4 |
| STM_DT2_42151 | rnr | SL4301 | ribonuclease R (RNase R) | 0 | PF08461.3 |
| STM_DT2_42271 | yjfP | SL4313 | conserved hypothetical protein | 0 | PF00326.14 |
| STM_DT2_42501 | ytfH | SL4335 | conserved hypothetical protein | 0 | PF01638.10 |
| STM_DT2_42501 | ytfH | SL4335 | conserved hypothetical protein | 0 | PF02872.11 |
| STM_DT2_42551 | ytfL | SL4340 | putative membrane protein | 0 | PF00571.21 |
| STM_DT2_42551 | ytfL | SL4340 | putative membrane protein | 0 | PF00571.21 |
| STM_DT2_42551 | ytfL | SL4340 | putative membrane protein | 0 | PF03471.10 |
| STM_DT2_43011 | treB | SL4385 | PTS system, trehalose-specific IIBC component (pseudogene) | 0 | PF00367.13 |
| STM_DT2_43151 | - | SL4399 | ornithine carbamoyltransferase | 0 | PF00185.17 |
| STM_DT2_43231 | - | SL4407 | cytosol aminopeptidase | 0 | PF02789.10 |
| STM_DT2_43251 | - | SL4409 | putative inner membrane protein | 0 | PF03739.7 |
| STM_DT2_43401 | - | SL4424 | type II restriction enzyme | 0 | PF02384.9 |
| STM_DT2_43631 | - | SL4446 | putative membrane protein | 0 | PF06779.7 |
| STM_DT2_43891 | mdoB | SL4472 | putative phosphoglycerol transferase | 0 | PF11575.1 |
| STM_DT2_43991 | - | SL4482 | putative membrane protein | 0 | PF00990.14 |
| STM_DT2_43991 | - | SL4482 | putative membrane protein | 0 | PF04972.10 |
| STM_DT2_44071 | - | SL4490 | conserved hypothetical protein | 0 | PF01734.15 |
| STM_DT2_44321 | creB | SL4515 | putative two-component response regulator | 0 | PF00072.17 |
| STM_DT2_34211 | glgP | SL3501 | glycogen phosphorylase | -0.1 | PF00343.13 |
| STM_DT2_06761 | - | SL0674 | lysR-family transcriptional regulator | -0.1 | PF00702.19 |
| STM_DT2_11291 | fabD | SL1131 | malonyl CoA-acyl carrier protein tran | -0.1 | PF00698.14 |
| STM_DT2_26371 | - | SL2662 | putative type I secretion protein | -0.1 | PF02321.11 |
| STM_DT2_43521 | - | SL4436 | hypothetical protein | -0.1 | PF01738.11 |
| STM_DT2_41121 | - | SL4198 | putative type-1 secretion protein | -0.1 | PF00005.20 |
| STM_DT2_32441 | oadA2 | SL3324 | oxaloacetate decarboxylase alpha chain | -0.1 | PF00364.15 |
| STM_DT2_01871 | yadB | SL0186 | glutamyl-tRNA synthetase-related prot | -0.1 | PF00749.14 |
| STM_DT2_12781 | - | SL1279 | conserved hypothetical protein | -0.1 | PF01326.12 |
| STM_DT2_24721 | guaB | SL2472 | inosine-5'-monophosphate dehydrogenase | -0.1 | PF00478.18 |
| STM_DT2_32171 | - | SL3297 | conserved hypothetical protein | -0.2 | PF10707.2 |
| STM_DT2_42511 | cpdB | SL4336 | 2',3'-cyclic-nucleotide 2'-phosphodiesterase | -0.2 | PF01595.13 |
| STM_DT2_25171 | hmpA | SL2518 | flavohemoprotein (haemoglobin-like protein) | -0.2 | PF00042.15 |
| STM_DT2_22741 | menE | SL2274 | O-succinylbenzoic acid-CoA ligase | -0.2 | PF00501.21 |
| STM_DT2_14211 | - | SL1424 | putative ABC transporter membrane protein | -0.3 | PF00528.15 |
| STM_DT2_01871 | yadB | SL0186 | glutamyl-tRNA synthetase-related prot | -0.3 | PF02834.9 |
| STM_DT2_18381 | argS | SL1844 | Arginyl-tRNA synthetase | -0.3 | PF00750.12 |
| STM_DT2_26961 | gabD | SL2775 | succinate-semialdehyde dehydrogenase | -0.3 | PF02867.8 |
| STM_DT2_36771 | - | SL3759 | putative carbohydrate kinase | -0.3 | PF06826.5 |
| STM_DT2_35231 | lpfC | SL3604 | outer membrane usher protein (LpfC) | -0.3 | PF00577.13 |
| STM_DT2_20791 | cpsB | SL2082 | mannose-1-phosphate guanylyltransferase | -0.4 | PF00483.16 |
| STM_DT2_09971 | - | SL1000 | conserved hypothetical protein | -0.4 | PF04403.6 |
| STM_DT2_25191 | cadB | SL2520 | probable cadaverine/lysine antiporter | -0.4 | PF00324.14 |
| STM_DT2_05671 | manX | SL0564 | putative mannose specific permease | -0.4 | PF03830.8 |
| STM_DT2_05761 | fepA | SL0573 | ferrienterobactin receptor precursor | -0.4 | PF00593.17 |
| STM_DT2_11511 | mfd | SL1153 | transcription-repair coupling factor | -0.4 | PF00270.22 |
| STM_DT2_31921 | - | SL3272 | Penicillin-binding protein (D-alanyl-D-alanine carboxypeptidase) | -0.4 | PF02113.8 |
| STM_DT2_25041 | iscS | SL2505 | Cysteine desulfurase | -0.5 | PF00266.12 |
| STM_DT2_34501 | livK | SL3530 | leucine-specific binding protein | -0.5 | PF01094.21 |
| STM_DT2_35851 | gpsA | SL3666 | glycerol-3-phosphate dehydrogenase | -0.5 | PF04127.8 |
| STM_DT2_07391 | - | SL0738 | putative hydro-lyase | -0.6 | PF05683.5 |
| STM_DT2_00381 | - | SL0038 | conserved hypothetical protein | -0.6 | PF03811.6 |
| STM_DT2_02551 | yafE | SL0254 | putative methyltransferase | -0.6 | PF06761.5 |
| STM_DT2_07461 | oadB2 | SL0745 | oxaloacetate decarboxylase beta chain | -0.6 | PF03977.6 |
| STM_DT2_31091 | cheM | SL3189 | methyl-accepting chemotaxis protein II | -0.6 | PF00202.14 |
| STM_DT2_06561 | ybeJ | SL0654 | ABC transporter periplasmic binding protein (glutamate/aspartate?) | -0.7 | PF03950.11 |
| STM_DT2_07631 | ybhE | SL0762 | conserved hypothetical protein | -0.7 | PF10282.2 |
| STM_DT2_40071 | rpoC | SL4093 | DNA-directed RNA polymerase, beta'-subunit | -0.7 | PF02844.8 |
| STM_DT2_21671 | mglB | SL2167 | D-galactose-binding periplasmic protein precursor | -0.7 | PF00532.14 |
| STM_DT2_33721 | damX | SL3452 | DamX protein | -0.7 | PF05036.6 |
| STM_DT2_01411 | yacF | SL0139 | conserved hypothetical protein | -0.7 | PF07072.4 |
| STM_DT2_18571 | otsB | SL1863 | trehalose phosphatase | -0.7 | PF02358.9 |
| STM_DT2_37981 | - | SL3886 | conserved hypothetical protein | -0.7 | PF07429.4 |
| STM_DT2_36721 | uhpC | SL3754 | regulatory protein | -0.8 | PF07690.9 |
| STM_DT2_09051 | ycaM | SL0906 | probable transport protein | -0.8 | PF00324.14 |
| STM_DT2_15741 | - | SL1581 | probable pyruvate-flavodoxin oxidoreductase | -0.8 | PF01558.11 |
| STM_DT2_15251 | sifB | SL1532 | putative virulence effector protein | -0.8 | PF06767.4 |
| STM_DT2_32441 | oadA2 | SL3324 | oxaloacetate decarboxylase alpha chain | -0.8 | PF10150.2 |
| STM_DT2_29791 | - | SL3059 | putative oxidoreductase | -0.8 | PF01276.13 |
| STM_DT2_00471 | ileS | SL0047 | isoleucyl-tRNA synthetase | -0.9 | PF00133.15 |
| STM_DT2_20051 | cbiD | SL2008 | Putative cobalt-precorrin-6A synthase | -0.9 | PF01888.10 |
| STM_DT2_23851 | xapA | SL2385 | xanthosine phosphorylase | -0.9 | PF01048.13 |
| STM_DT2_00891 | folA | SL0088 | dihydrofolate reductase type I | -0.9 | PF00186.12 |
| STM_DT2_39521 | menG | SL4038 | menaquinone biosynthesis protein | -0.9 | PF03737.8 |
| STM_DT2_04561 | ybaO | SL0453 | hypothetical transcriptional regulator | -1 | PF01037.14 |
| STM_DT2_10351 | hpaB | SL1038 | 4-hydroxyphenylacetate 3-monooxygenas | -1 | PF03241.6 |
| STM_DT2_14641 | hyaB2 | SL1467 | uptake hydrogenase-1 large subunit | -1 | PF00374.12 |
| STM_DT2_27821 | sipD | SL2862 | pathogenicity island 1 effector protein | -1 | PF05192.11 |
| STM_DT2_42911 | - | SL4375 | hypothetical protein | -1 | PF01979.13 |
| STM_DT2_16501 | trpB | SL1657 | tryptophan synthase beta chain | -1.2 | PF00291.18 |
| STM_DT2_04971 | - | SL0494 | putative membrane protein | -1.2 | PF01145.18 |
| STM_DT2_43231 | - | SL4407 | cytosol aminopeptidase | -1.3 | PF00883.14 |
| STM_DT2_11881 | - | SL1191 | putative substrate-binding transport protein | -1.4 | PF00496.15 |
| STM_DT2_14111 | - | SL1414 | putative secreted protein | -1.4 | PF00089.19 |
| STM_DT2_03891 | yajF | SL0388 | hypothetical ROK-family protein | -1.4 | PF02903.7 |
| STM_DT2_30351 | - | SL3115 | hypothetical protein | -1.4 | PF00939.12 |
| STM_DT2_12781 | - | SL1279 | conserved hypothetical protein | -1.5 | PF02696.7 |
| STM_DT2_15921 | - | SL1599 | invasin-like protein | -1.5 | PF11924.1 |
| STM_DT2_39121 | rhaR | SL3998 | L-rhamnose operon transcriptional activator | -1.5 | PF02518.19 |
| STM_DT2_26311 | recN | SL2656 | DNA repair protein | -1.6 | PF02321.11 |
| STM_DT2_23731 | - | SL2373 | putative decarboxylase | -1.7 | PF00205.15 |
| STM_DT2_18591 | yecI | SL1865 | ferritin-like protein | -1.8 | PF00210.17 |
| STM_DT2_00221 | bcfB | SL0022 | fimbrial chaperone | -2 | PF00345.13 |
| STM_DT2_24841 | gcpE | SL2485 | 4-hydroxy-3-methylbut-2-en-1-yl diphosphate synthase | -2 | PF04551.7 |
| STM_DT2_42601 | - | SL4345 | putative sugar transporter | -2.1 | PF07690.9 |
| STM_DT2_13761 | - | SL1378 | conserved hypothetical protein | -2.1 | PF00730.18 |
| STM_DT2_07191 | sucA | SL0718 | 2-oxoglutarate dehydrogenase E1 compo | -2.1 | PF08442.3 |
| STM_DT2_08121 | ybiR | SL0811 | putative membrane protein | -2.3 | PF03600.9 |
| STM_DT2_06701 | glnS | SL0668 | glutaminyl-tRNA synthetase | -2.4 | PF03466.13 |
| STM_DT2_08441 | - | SL0844 | putative membrane protein | -2.5 | PF07690.9 |
| STM_DT2_34271 | gntU | SL3507 | low-affinity gluconate transporter | -2.5 | PF02447.9 |
| STM_DT2_24741 | shdA | SL2474 | host colonisation factor (ShdA) | -2.6 | PF05689.4 |
| STM_DT2_42931 | - | SL4377 | conserved hypothetical protein | -2.6 | PF02378.11 |
| STM_DT2_27461 | hycI | SL2825 | hydrogenase 3 maturation protease | -2.7 | PF01750.11 |
| STM_DT2_10431 | hpaX | SL1046 | putative 4-hydroxyphenylacetate permease | -3 | PF07690.9 |
| STM_DT2_18431 | flhB | SL1849 | flagellar biosynthetic protein FlhB | -3 | PF01312.12 |
| STM_DT2_04901 | ushA | SL0487 | UDP-sugar hydrolase | -3.1 | PF00149.21 |
| STM_DT2_35631 | - | SL3644 | conserved hypothetical protein | -3.2 | PF12390.1 |
| STM_DT2_05191 | - | SL0517 | putative permease protein | -3.4 | PF00860.13 |
| STM_DT2_05791 | entF | SL0576 | enterobactin synthetase component F | -3.8 | PF00501.21 |
| STM_DT2_20451 | hisG | SL2048 | ATP phosphoribosyltransferase | -3.8 | PF01634.11 |
| STM_DT2_42601 | - | SL4345 | putative sugar transporter | -4.2 | PF09863.2 |
| STM_DT2_25801 | - | SL2581 | putative prophage protein | -4.4 | PF05766.5 |
| STM_DT2_12871 | - | SL1288 | putative electron transfer flavoprotein subunit | -5 | PF01012.14 |
